# Synonymous mutations make dramatic contributions to fitness when growth is limited by a weak-link enzyme

**DOI:** 10.1371/journal.pgen.1007615

**Published:** 2018-08-27

**Authors:** JohnCarlo Kristofich, Andrew B. Morgenthaler, Wallis R. Kinney, Christopher C. Ebmeier, Daniel J. Snyder, William M. Old, Vaughn S. Cooper, Shelley D. Copley

**Affiliations:** 1 Department of Molecular, Cellular and Developmental Biology, University of Colorado Boulder, Boulder, CO, United States of America; 2 Cooperative Institute for Research in Environmental Sciences, University of Colorado Boulder, Boulder, CO, United States of America; 3 University of Pittsburgh Medical Center, Pittsburgh, Pennsylvania, United States of America; Université Paris Descartes, INSERM U1001, FRANCE

## Abstract

Synonymous mutations do not alter the specified amino acid but may alter the structure or function of an mRNA in ways that impact fitness. There are few examples in the literature, however, in which the effects of synonymous mutations on microbial growth rates have been measured, and even fewer for which the underlying mechanism is understood. We evolved four populations of a strain of *Salmonella enterica* in which a promiscuous enzyme has been recruited to replace an essential enzyme. A previously identified point mutation increases the enzyme’s ability to catalyze the newly needed reaction (required for arginine biosynthesis) but decreases its ability to catalyze its native reaction (required for proline biosynthesis). The poor performance of this enzyme limits growth rate on glucose. After 260 generations, we identified two synonymous mutations in the first six codons of the gene encoding the weak-link enzyme that increase growth rate by 41 and 67%. We introduced all possible synonymous mutations into the first six codons and found substantial effects on growth rate; one doubles growth rate, and another completely abolishes growth. Computational analyses suggest that these mutations affect either the stability of a stem-loop structure that sequesters the start codon or the accessibility of the region between the Shine-Dalgarno sequence and the start codon. Thus, these mutations would be predicted to affect translational efficiency and thereby indirectly affect mRNA stability because translating ribosomes protect mRNA from degradation. Experimental data support these hypotheses. We conclude that the effects of the synonymous mutations are due to a combination of effects on mRNA stability and translation efficiency that alter levels of the weak-link enzyme. These findings suggest that synonymous mutations can have profound effects on fitness under strong selection and that their importance in evolution may be under-appreciated.

## Author summary

When a new enzyme is needed, microbes often recruit a pre-existing enzyme with a promiscuous activity corresponding to the newly needed activity. Such enzymes are often the “weak-link” in metabolism because they have not evolved to efficiently catalyze the new reaction. Under these circumstances, increasing the level of the weak-link enzyme can improve fitness. We evolved a strain of *S*. *enterica* in which a weak-link enzyme–E383A ProA–serves essential functions in synthesis of proline and arginine for 260 generations and then sequenced the genomes of several evolved strains. A mutation in the promoter of the operon encoding E383A ProA increased growth rate 9-fold. More surprisingly, a mutation upstream of the start codon and two synonymous mutations within the first six codons also increased growth rate by up to 68%. Introduction of all possible synonymous mutations in the first six codons showed that some doubled growth rate, while others slowed or even prevented growth. Computational and experimental data suggest that these effects were due to enhanced translational efficiency of the weak-link enzyme. These results show that synonymous mutations, once assumed to be selectively neutral, can have strong impacts on fitness when growth rate is limited by a weak-link enzyme.

## Introduction

Synonymous mutations have traditionally been considered to be silent with respect to fitness because they do not change the encoded amino acid. However, synonymous mutations can alter mRNA structures in ways that alter translation initiation, mRNA stability, or even protein folding due to changes in the tempo of translation. Codon choice is not necessarily optimized in every gene, as natural selection operates to favor only mutations that impact survival, growth and/or reproduction, and even detrimental mutations can be fixed when population sizes are small or population bottlenecks occur. Nevertheless, codon choice appears to be reasonably close to optimal under the normal conditions in which organisms grow and reproduce, and synonymous mutations under those circumstances are often detrimental. Indeed, synonymous mutations have been implicated in over 50 human diseases [1], including cystic fibrosis, breast cancer [2], melanoma [3] and Crohn’s disease [4]. The same holds true for microbes; most of the 38 synonymous mutations introduced into genes encoding the *E*. *coli* ribosomal proteins S20 and L1 were slightly deleterious, with an average selection coefficient of -0.0096 [5].

While most synonymous mutations are neutral or slightly detrimental under normal conditions [6], their effects may be magnified under strong selection. Synonymous mutations that generate a new promoter for a critical enzyme encoded by a downstream gene have been reported. In *E*. *coli*, for example, a synonymous mutation in the gene upstream of *inhA*, which encodes the target of isoniazid (used to treat tuberculosis), generates a new promoter and increases *inhA* expression by 3-4-fold [7]. A similar case is our discovery of a synonymous mutation in the gene upstream of a gene encoding an inefficient bifunctional enzyme; this mutation also generates a new promoter for the downstream gene [8, 9].

The effects of synonymous mutations can also play out at the mRNA level. Synonymous mutations can influence the stability of helices and stem-loops in ways that affect translation efficiency, mRNA stability or both. Synonymous mutations can also affect recognition of mRNAs by small regulatory RNAs and even the tempo of translation by the ribosome [10]. An infrequently used codon recognized by a rare tRNA can cause a pause in translation and allow the polypeptide to fold in the absence of interference from downstream sequences [11]. Thus, synonymous mutations can lead to different folding pathways and consequent effects on protein function [12–15].

**Fig 1:**
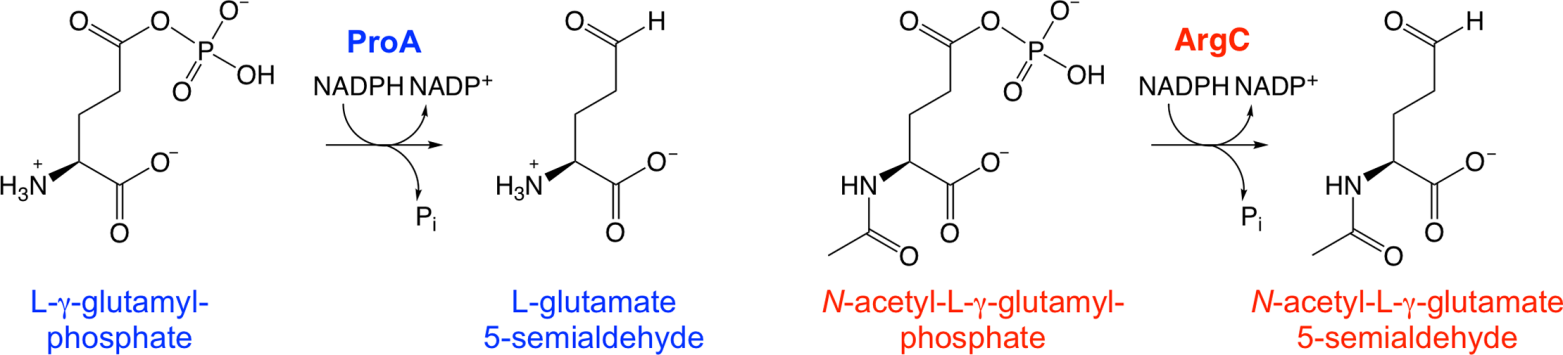
The reactions catalyzed by ProA (γ-glutamyl phosphate reductase) and ArgC (N-acetyl glutamyl phosphate reductase).

**Fig 2:**
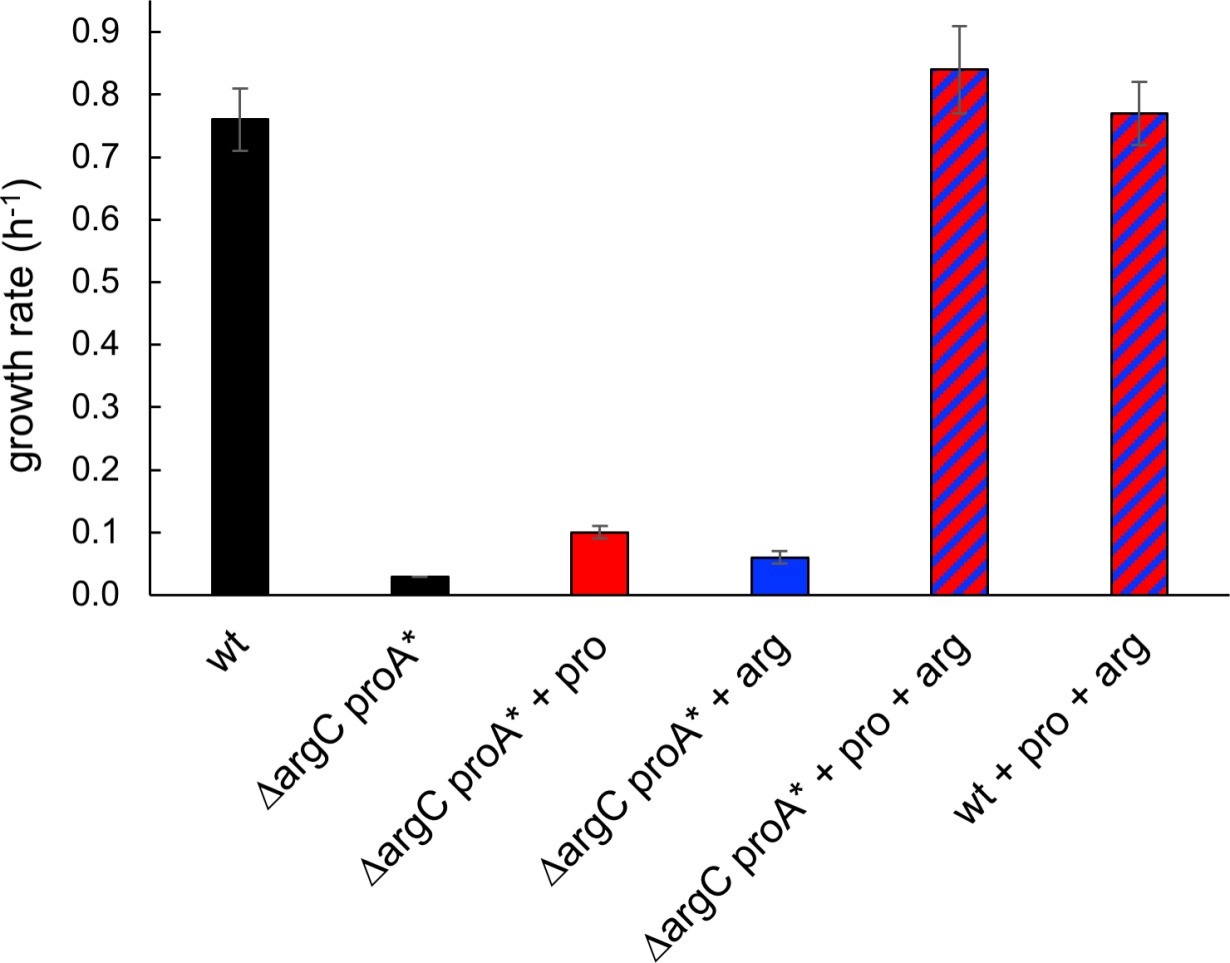
Restoration of growth rate of the ΔargC proA* strain to the level of the wild-type strain requires addition of both proline (0.4 mM) and arginine (5.2 mM).

Here we report strikingly large effects–both beneficial and detrimental–of synonymous mutations in a gene encoding a “weak-link” enzyme that catalyzes two reactions essential for growth of a mutant strain of *Salmonella enterica* subsp. *enterica* serovar Typhimurium *str*. SL1344 in M9/glucose. ProA (L-γ-glutamyl phosphate reductase) has a promiscuous ability to handle the substrate for ArgC (*N*-acetyl-L-glutamyl phosphate reductase) (Fig 1). This activity is not sufficient to sustain growth when *argC* is deleted from *Salmonella enterica*. However, a point mutation that changes Glu382 to Ala allows ProA to catalyze both reactions. (This mutation is analogous to the mutation that enables survival of Δ*argC E*. *coli* on glucose that we previously described [8].) The inefficiency of E382A ProA (referred to as ProA* hereafter) severely limits growth of ΔargC proA* S. *enterica* on M9/glucose (Fig 2), imposing strong selective pressure for emergence of mutants that have an improved ability to synthesize proline and arginine.

We evolved four populations founded by the ΔargC proA* strain under conditions selecting for faster growth for 260 generations. A mutation in the -10 region of the promoter of the proBA* operon that increases growth rate by 8-fold appeared first in every lineage. Subsequently, we detected an intergenic mutation just upstream of proA*, two synonymous mutations within the first six codons of proA* and one non-synonymous mutation in proA*. When re-introduced into the ΔargC proA* strain carrying the promoter mutation, these mutations increased growth rate by 30–83%. The striking effects of the two synonymous mutations inspired us to introduce all possible synonymous mutations in the first six codons of the proA* mRNA. Among these mutations, one doubled growth rate, while another completely abolished growth on M9/glucose. These fitness effects exceed previously reported effects for synonymous mutations [6, 16, 17].

Growth rate in strains containing the promoter mutation and mutations near the head of proA* (the second gene in the proBA* operon) correlates with changes in the level of proA* mRNA, suggesting that the mutations impact mRNA stability. Computational analyses of the structures of this region of wild-type and mutant *proA* mRNAs suggest that the mutations alter the efficiency of translation initiation, a conclusion supported by the observation that the mutations cause larger fold-changes in ProA* levels than in proA* mRNA levels. Thus, we attribute the striking fitness effects of synonymous mutations in the head region of the proA* mRNA to increases in production of ProA* due to both an increase in the level of proA* mRNA and an increase in the amount of protein produced from each mRNA molecule.

## Results

### Growth of the ΔargC proA* strain is impaired by a poor ability to synthesize both proline and arginine

The genesis of this project was an interest in how recruitment of a promiscuous enzyme to serve a new function and the subsequent process of gene duplication and divergence to alleles encoding two specialist enzymes might differ in different organisms. We previously found that a point mutation in *proA* allows E383A ProA to substitute for ArgC in *E*. *coli*. In this work, we deleted *argC* from *Salmonella enterica* subsp. *enterica* serovar Typhimurium *str*. SL1344 and introduced a point mutation changing Glu382 (which is homologous to Glu383 in *E*. *coli*) to Ala. The inefficiency of E382A ProA (ProA*) limits growth rate to only 4% that of wild-type cells (Fig 2). Full restoration of growth requires addition of both proline and arginine, indicating that ProA* is the weak-link enzyme limiting growth rate of the ΔargC proA* strain, and that both L-γ-glutamylphosphate reductase and *N*-acetyl-L-γ-glutamylphosphate reductase activities are insufficient for optimal growth in this strain.

### Identification of mutations in the proBA* operon in large colonies of the ΔargC proA* strain on M9/glucose plates

**Fig 3:**
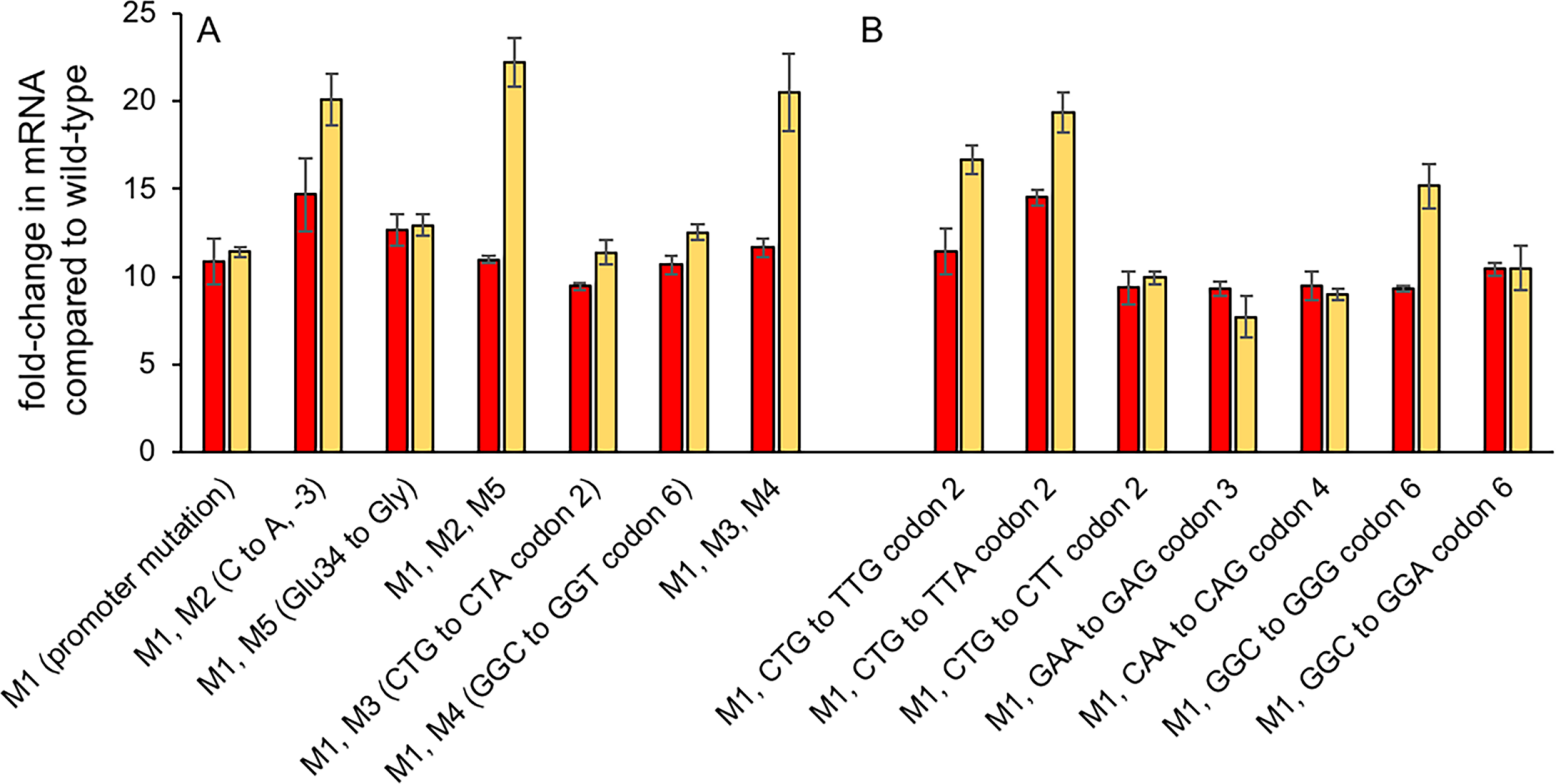
The effects of point mutations in the proBA* operon and near the head of the proA* mRNA discovered on the levels of *proB* (red) and proA* (gold) mRNA in strains carrying mutations discovered in the adaptive evolution experiment (A) and in strains with additional synonymous mutations created by genome editing (B). Error bars indicate 1 SD.

When the ΔargC proA* strain was spread onto plates containing M9/glucose, we observed a few large colonies among a background of small colonies (S1 Fig), suggesting that cells in some colonies had acquired a mutation that increased growth rate. In previous experiments with the comparable system in *E*. *coli*, 9% of the large colonies contained amplifications of a region surrounding the proBA* operon [9]. We assessed the copy number of the proBA* operon in 100 large colonies of the ΔargC proA* S. *enterica* strain but detected no amplifications. We sequenced the proBA* operon in 50 of these colonies and detected a mutation in the -10 region of the promoter in each colony. (*proB* encodes γ-glutamyl kinase, which synthesizes the substrate for ProA.) This mutation (M1), which changes the -10 region of the promoter from TAAAAC to TAAAAT, increased the levels of both *proB* and proA* mRNA by about 11-fold (Fig 3A).

### Evolution of the ΔargC proA* strain in M9/glucose

**Fig 4:**
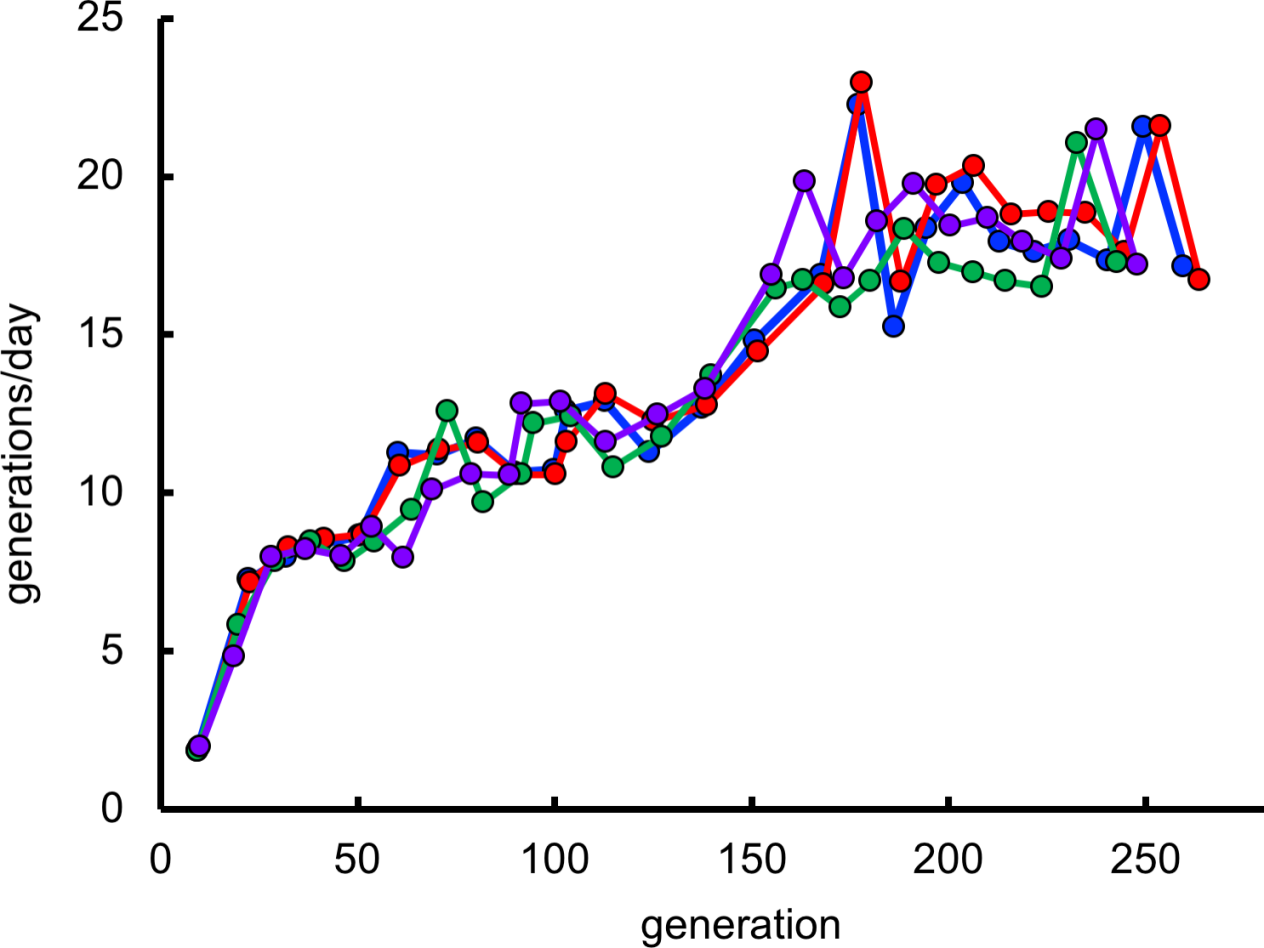
Improvement of growth rate during evolution of four populations of the ΔargC proA* strain on M9/glucose.

**Fig 5:**
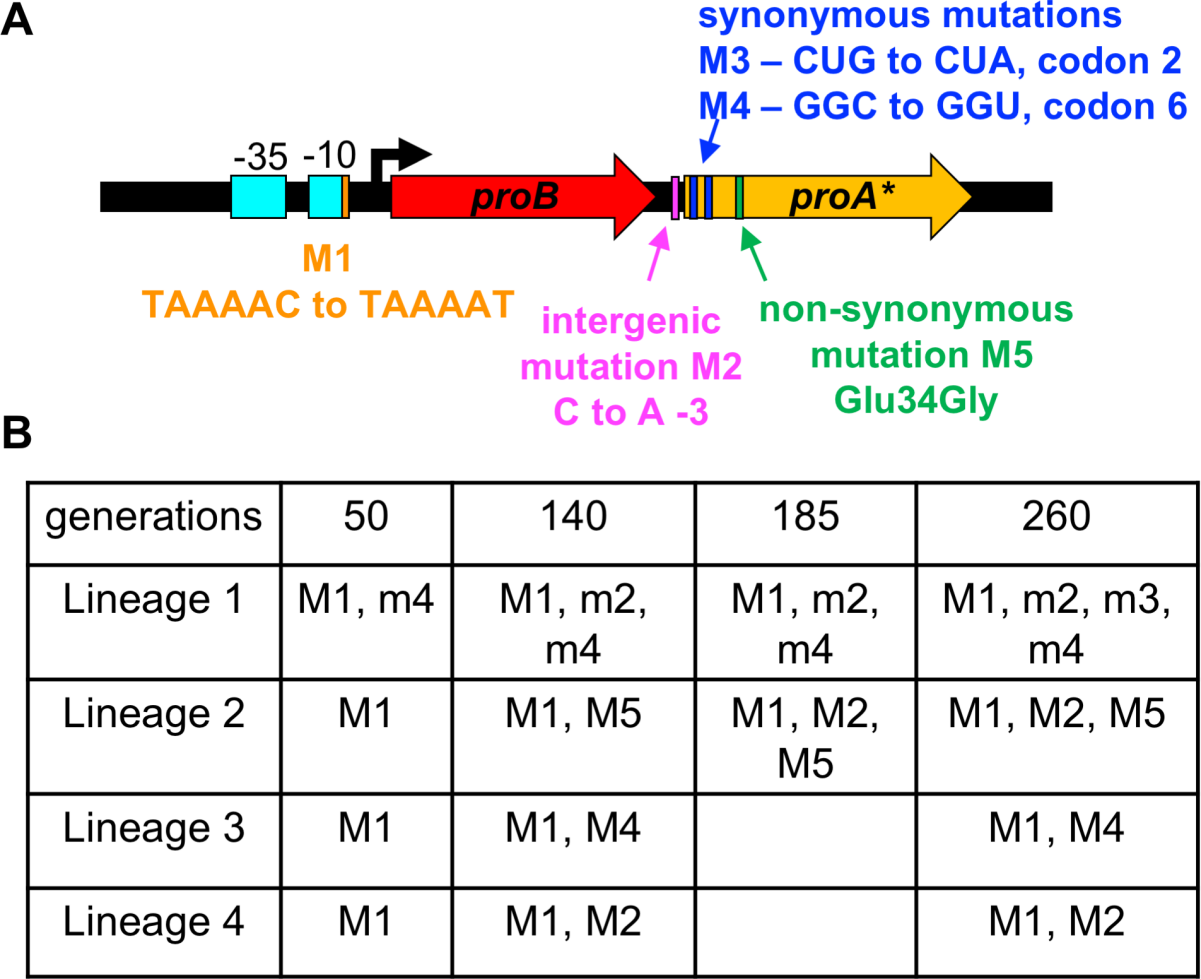
Mutations detected in the proBA* operon in four lineages of the ΔargC proA* strain after adaptation for approximately 250 generations in M9/glucose. A) Locations of point mutations; B) presence of point mutations in the population at the indicated time points. Lower-case letters indicate that the mutation was present in a fraction of the population based on Sanger sequencing. Each of these mutations was subsequently detected in individual colonies from the population, as well. Upper-case letters denote mutations that had swept the population to the limit of detection by Sanger sequencing.

We evolved four populations of the **Δ*argC*** proA* S. *enterica* strain in M9/glucose for approximately 260 generations (Fig 4). Growth rate, as approximated by generations per day, increased 8-10-fold over this period. We measured the copy number of proA* by qPCR every 60 generations, but no amplification was detected. We sequenced the proBA* operon by Sanger sequencing (which will only detect the most abundant clones) in genomic DNA isolated from each population at 3–4 points during the evolution experiment. In addition to the promoter mutation M1 previously identified in large colonies on plates, we identified an intergenic mutation at -3 relative to the proA* start codon, synonymous mutations in codons 2 and 6 of proA*, and a non-synonymous mutation in codon 34 of proA* (Fig 5A). Fig 5B shows the mutations detected in the proBA* operon in each population at various times during adaptation. M1 occurred first in each lineage. Subsequently, 1–3 additional mutations were found in each lineage. Sequencing of the proBA* operon in individual colonies revealed that M1 occurred with each of the other mutations. Colonies with more than two mutations had either M1, M2 and M5, or M1, M3 and M4.

### Whole-genome sequencing of adapted clones

We re-sequenced the genomes of the parental strain and of 7 colonies isolated from the final populations to determine whether the fitness increase during adaptation resulted from mutations in the proBA* operon or elsewhere in the genome (Table 1). All colonies, as well as the parental strain, contained identical mutations in *menC*, *manX*, *ybdN*, and SL1344_2940 compared to the reference genome. *menC* encodes an enzyme involved in menaquinone biosynthesis, which is primarily required for anaerobic growth [18]. *manX* encodes a mannose-specific phosphotransferase IIAB component. Since all of our experiments were carried out under aerobic conditions and with glucose as a sole carbon source, these mutations likely had little effect on growth rate. The other two genes in which mutations were found are genes of unknown function.

**Table 1:** Mutations found in seven adapted clones.

strain	lineage	mutations in proBA* operon	other mutations^a^
JC665	1	M1, M3, M4	
JC667	1	M1, M2	6 bp deletion of CGATCG in (CGATCG)_2_ at 546 in *cmk* (encodes cytidylate kinase)
JC669	2	M1, M2, M5	
JC671	2	M1, M2, M5	
JC673	3	M1, M4	C -> A in *speF* changes Ala657 to Asp
			1 bp deletion at nucleotide 313 in *cmk;* results in premature stop codon
JC675	3	M1, M4	C -> A in *speF* changes Ala657 to Asp
			1 bp deletion at nucleotide 313 in *cmk*; results in pre-mature stop codon
JC677	4	M1, M2	C -> A in *speF* changes Ala657 to Asp
			22 bp deletion at nucleotide 538 in *cmk*; results in pre-mature stop codon

^a^in addition to mutations found in the parental strain

The genome sequences of two colonies from population 2 were identical, as were those of two colonies from population 3. Deletions in *cmk* were found in one colony from population 1, both colonies from population 3, and the single colony from population 4. A point mutation in *speF* was found in population 3 and 4. The parallelism in mutation targets among the small number of lineages strongly suggests that these mutations contributed to fitness during the adaptation. *speF* encodes ornithine decarboxylase. Ornithine is an intermediate in arginine biosynthesis downstream of the reaction catalyzed by the weak-link ProA*. Decreasing ornithine decarboxylase activity, which diverts ornithine toward putrescine synthesis, might prevent diversion of material from the compromised arginine synthesis pathway. *cmk* encodes cytidylate kinase, which is involved in pyrimidine salvage. Two of the three observed mutations introduced stop codons and likely caused loss of function. It is not obvious why loss of function of cytidylate kinase would be useful under these selection conditions.

### Each point mutation found in the proBA* operon enhances fitness

We introduced each of the point mutations identified in the proBA* operon, as well as combinations of mutations observed after the conclusion of the adaptation, back into the ΔargC proA* strain and re-sequenced the genomes of the constructed strains to identify any mutations acquired during genome editing (Table 2). The strain in which M1 had been introduced (JC559) had an additional mutation near the 3’-end of *glnD*. GlnD encodes a uridyl transferase/uridyl removing enzyme that transfers a uridyl group to PII under nitrogen-limiting conditions, leading to activation of glutamine synthetase. Since each of the reconstructed strains contains this mutation, the effects of the additional introduced mutations can be evaluated in a common genetic background. Two other reconstructed strains had acquired additional mutations. The strain containing M1 and M5 (JC622) had a deletion in *bssS*, a gene involved in biofilm formation. BssS was not among the 1607 proteins detected by proteomic analyses of samples grown under planktonic conditions; thus, a deletion in *bssS* should not affect fitness. The strain containing M1, M3 and M4 (JC663) had a point mutation in *napH*. NapH is one component of the NapGH quinol dehydrogenase that is involved in nitrate reduction under anaerobic conditions. Because there is no nitrate in M9/glucose, this mutation should not affect planktonic growth in M9/glucose under aerobic conditions.

**Table 2:** Strains used in this work.

strain	genotype	notes
JK411	*Salmonella enterica* subsp. *enterica serovar* Typhimurium *str*. SL1344; +*hisG* (HisG P69L); L148L MenC (TTA->TTG); ManX E95V (GAG->GTG)	initial strain in which the mutation causing histidine auxotrophy had been reverted; carries unexpected mutations in *menC and manX*
JK328	JK411 Δ*argC*; proA* (E382A ProA); SL1344_2940 V333A (GTT->GCC); YbdN T381I (ACA->ATA)	"parental strain"; mutations in SL1344_2940 and *ybdN*
JC559	JK328 + M1 (C365750T); GlnD D837V (GAT->GTT)	contains a mutation in *glnD*
JC592	JC559 + M4 (C366925T)	
JC596	JC559 + M3 (G366913A)	
JC620	JC559 + M2 (C366905A)	
JC622	JC559 + M5 E34G ProA (GAA->GGA); Δ8bp *bssS* (+68–75)	deletion in *bssS*, a gene involved in biofilm formation
JC649	JC559 + M2 (C366905A) and M5 E34G ProA (GAA->GGA)	
JC663	JC559 + M3 (G366913A) and M4 (C366925T); NapH A12E (GCG->GAG)	mutation in *napH*, a gene involved in nitrate reduction
WK012	JC559—M1 + M4	
WK014	JC559—M1 + M3	mutation that changes Gly95 to Ser in SL1344_2574, a putative bacteriophage protein
JC706	JC559 + C4T (C366931T) and G6A *proA* (G366933A)	
JC710	JC559 + C4T *proA* (C366931T)	
JC712	JC559 + A9G *proA* (A366936G)	
JC716	JC559 + C18G *proA* (C366945G); LtaE S97S (AGC->AGT)	synonymous mutation in *ltaE*
JC718	JC559 + G6T *proA* (G366933T)	
JC720	JC559 + G6C *proA* (G366933C)	
JC722	JC559 + A12G *proA* (A366939G)	
JC724	JC559 + C18A *proA* (C366945A)	

**Fig 6:**
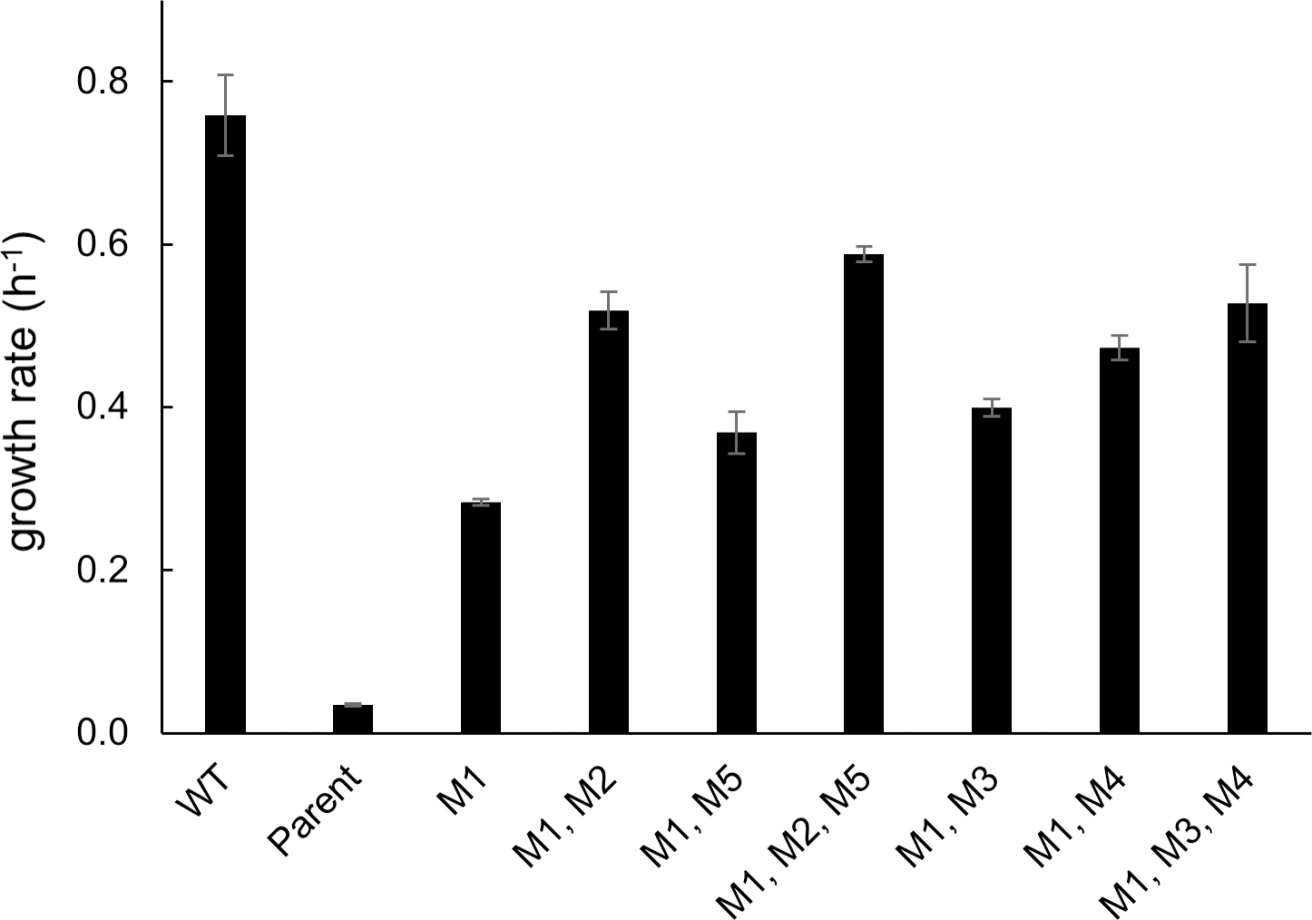
Comparison of the growth rates of the ΔargC proA* strain in which the indicated mutations had been introduced by genome editing. Error bars represent 1 SD.

Fig 6 shows the growth rates of the reconstructed strains. The promoter mutation M1 increases growth rate by 9.2-fold over that of the parental ΔargC proA* strain. In the background of M1, each of the additional point mutations further increases growth rate by factors ranging from 1.3- to 1.8-fold. The combinations of M2 and M5 and of M3 and M4 observed at the end of the evolution experiment provided further increases in growth rate, resulting in strains with a 17-18-fold improvement over the growth rate of the parental strain as a result of only three mutations. We also introduced the synonymous mutations M3 and M4 into the parental strain in the absence of the promoter mutation M1. Both mutations increased growth rate less in the presence of M1 than in the parental background (S2 Fig). In the parental background, M3 and M4 conferred a 2.1- and 4.0-fold increase in growth rate, respectively, while they conferred only a 1.4- and 1.7-fold increase in growth rate in the M1 background. These negative epistatic effects indicate that the increase in ProA* due to the synonymous mutations is less impactful after the growth limitation has been partially solved by the promoter mutation M1.

### Other synonymous mutations in the first six codons have substantial effects on growth rate

The occurrence of synonymous mutations in codons 2 and 6 of proA* that increased growth rate by 1.4- and 1.7-fold, respectively, in the background of the promoter mutation M1 prompted us to ask whether other synonymous mutations in this region would affect fitness. We introduced all possible synonymous mutations into codons 2,3,4 and 6. (Codons 1 and 5 specify Met, and there is only one codon choice for this amino acid.) The whole genome of each constructed strain was sequenced to identify any adventitious mutations introduced during genome editing (Table 2). Fig 3B shows the effects of these mutations on the levels of *proB* and proA* mRNAs in the ΔargC proA* strain containing the promoter mutation M1.

**Fig 7:**
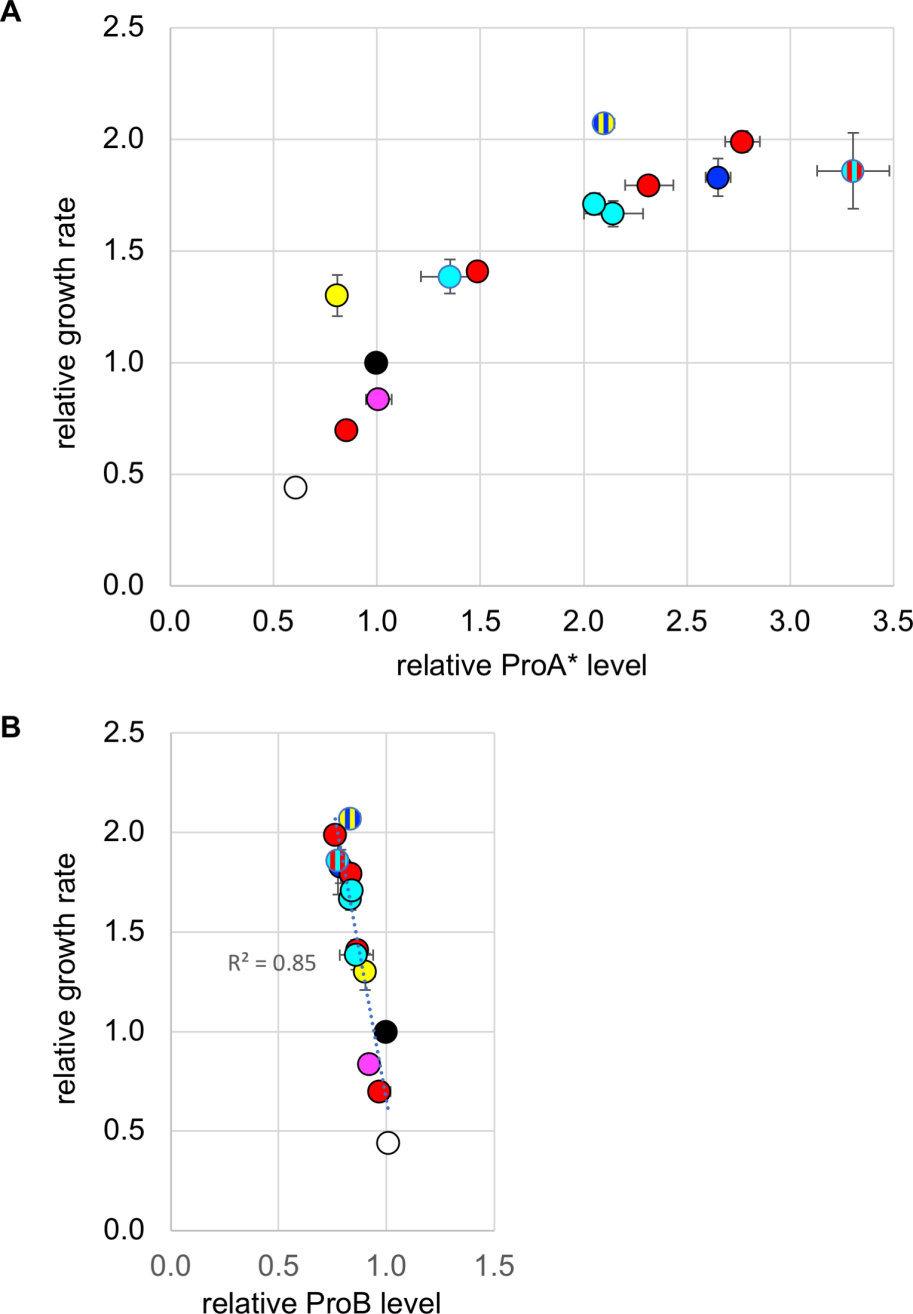
Growth rates and levels of ProA* (A) and ProB (B) in strains carrying the promoter mutation M1 and other mutations relative to those of the strain carrying just M1. Black, no other mutations in head region; blue, intergenic mutation M2; red, synonymous mutations in codon 2; white, synonymous mutation in codon 3; magenta, synonymous mutation in codon 4; cyan, synonymous mutations in codon 6; yellow, non-synonymous mutation M5. Data points representing strains with M1 and two additional mutations are shown with colored stripes corresponding to the colors used for individual mutations. Error bars represent 1 SD.

Because mRNA levels are often not well-correlated with protein levels, we measured the abundances of ProA* and ProB in strains containing promoter mutation M1 and synonymous mutations relative to those in the strain containing just M1 (S1 Table) using label-free high resolution Orbitrap mass spectrometry [19]. Fig 7 shows that ProA* levels varied over a 5.4-fold range, whereas ProB levels varied by only 20%. Growth rate increases as a function of the level of ProA*, leveling off at 2-fold higher at the highest levels of the protein. In contrast, there is a surprising anti-correlation between growth rate and the level of ProB. Whether a decrease in ProB level contributes to fitness independently of an increase in ProA* level is uncertain.

### Prediction of the secondary structures for the region surrounding the Shine-Dalgarno sequence and start codon for wild-type and mutant versions of proA*

We utilized the RNAstructure package (version 5.8.1) (https://rna.urmc.rochester.edu/RNAstructureWeb/) [20] with the default parameters to predict the secondary structures of the head region of the proA* mRNA. The choice of the fragment length for the modeling was considered carefully. mRNA will be exposed behind a translating ribosome and will begin to fold as soon as base pair interactions are possible. Because the ribosome occludes about 11 codons of mRNA [21], a new initiation cycle cannot be started until the preceding ribosome moves at least 11 codons away from the start codon. Thus, we chose to model the structures of 53-nucleotide mRNA fragments beginning 4 nucleotides upstream of the Shine-Dalgarno sequence and continuing 34 nucleotides past the AUG start codon.

**Fig 8:**
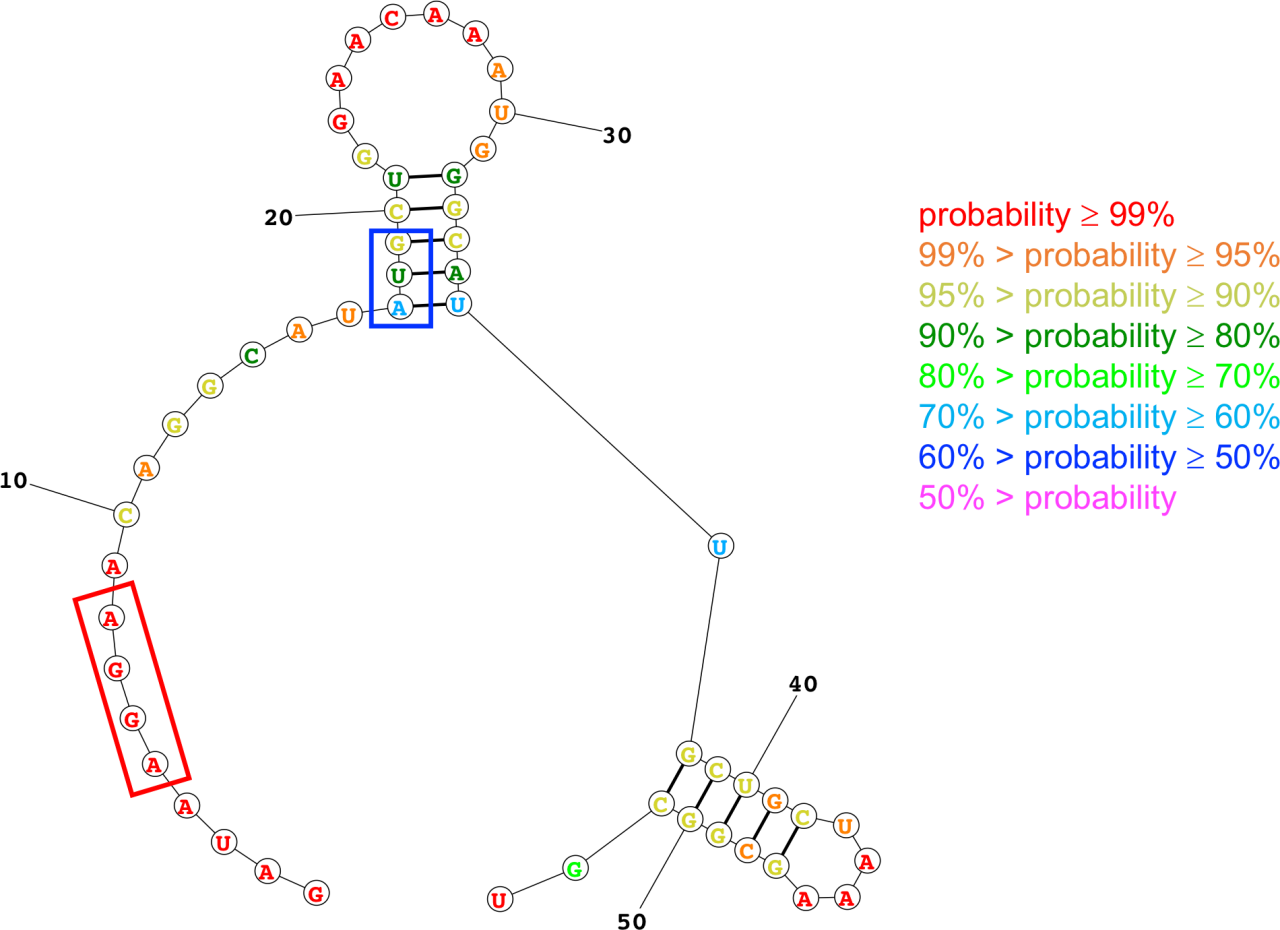
Predicted lowest-free energy structure of the 53-nt fragment of the head of the wild-type proA* transcript generated by the Fold algorithm. The Shine-Dalgarno sequence is indicated by the red box and the start codon by the blue box. The colors of the bases indicate the probability that each base is found in the depicted state.

The lowest-energy structure for the 53-nucleotide region of the proA* mRNA generated by the Fold algorithm is shown in Fig 8. The color used for each nucleotide conveys the probability that the nucleotide is found in the depicted state. The Shine-Dalgarno sequence [22] is predicted to be single-stranded and therefore accessible for binding to the 30S subunit of the ribosome in this structure, as well as all of the mutant structures to be discussed below. Notably, the AUG start codon is sequestered in a 5-bp stem that includes parts of codons 2 and 7 and all of codon 6. The mutations discussed below do not alter the structure of the second stem-loop at the lower right of Fig 8A, so this part of the structure will not be shown in the following figures. Minimal free energies for each calculated structure are shown in Table 3.

**Table 3:** Effects of mutations on growth rate relative to the parental strain containing the promoter mutation M1 and the minimal folding energy of 53-nucleotide fragments surrounding the proA* start codon^a^.

mutations	fold-change in growth rate	minimal folding energy (kcal/mol)
**M1** only	1	-12.3
**M1**, **M2** (C to A -3)	1.84 (0.08)^b^	-12.3
**M1, M5**	1.32 (0.09)	-12.3
**M1, M2, M5**	2.07 (0.04)	-12.3
**M1**, codon 2 CUG to UUG	1.80 (0.03)	-9.8
**M1**, codon 2 CUG to UUA	1.99 (0.05)	-9.3
**M1**, codon 2 from CUG to CUA (**M3**)	1.41 (0.04)	-11.8
**M1**, codon 2 CUG to CUU	0.70 (0.02)	-13.0
**M1**, codon 2 CUG to CUC	0	-15.2
**M1**, codon 3 GAA to GAG	0.44 (0.02)	-12.3
**M1**, codon 4 CAA to CAG	0.84 (0.02)	-12.3
**M1**, **M4 (**codon 6 GGC to GGU)	1.67 (0.06)	-10.3
**M1, M3, M4**	1.86 (0.17)	-9.8
**M1**, codon 6 GGC to GGG	1.71 (0.05)	-9.4
**M1**, codon 6 GGC to GGA	1.40 (0.04)	-9.4

^a^ Bold indicates mutations observed in the adapted strains.

^b^ Numbers in parentheses indicate 1 SD.

**Fig 9:**
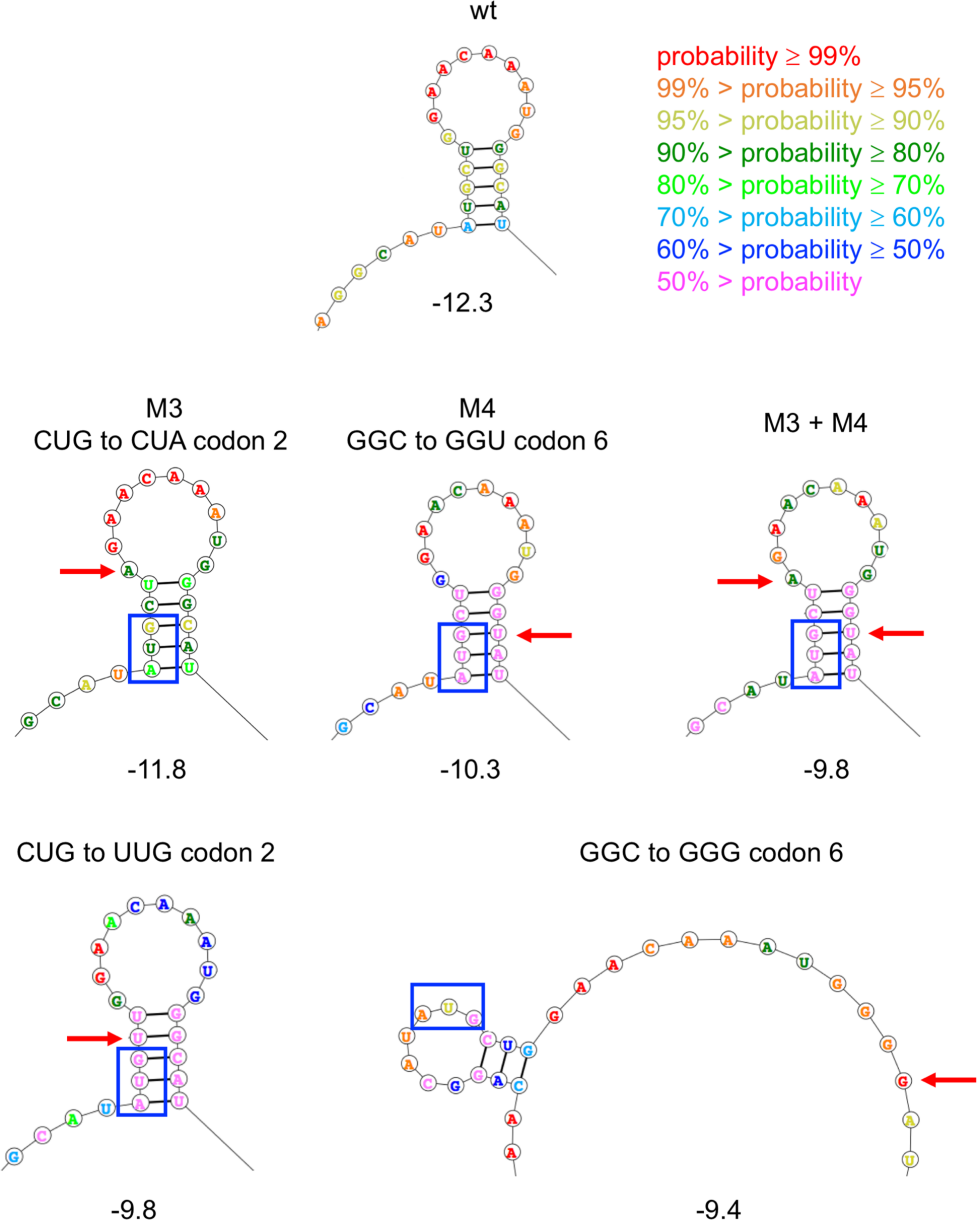
Predicted lowest-free energy structures of the region surrounding the AUG start codon (blue box) in wild-type and in mutant proA* transcripts in which synonymous mutations in codons 2 and 6 increase growth rate. Numbers underneath the structures indicate the folding energy calculated by the Fold algorithm in kcal/mol.

**Fig 10:**
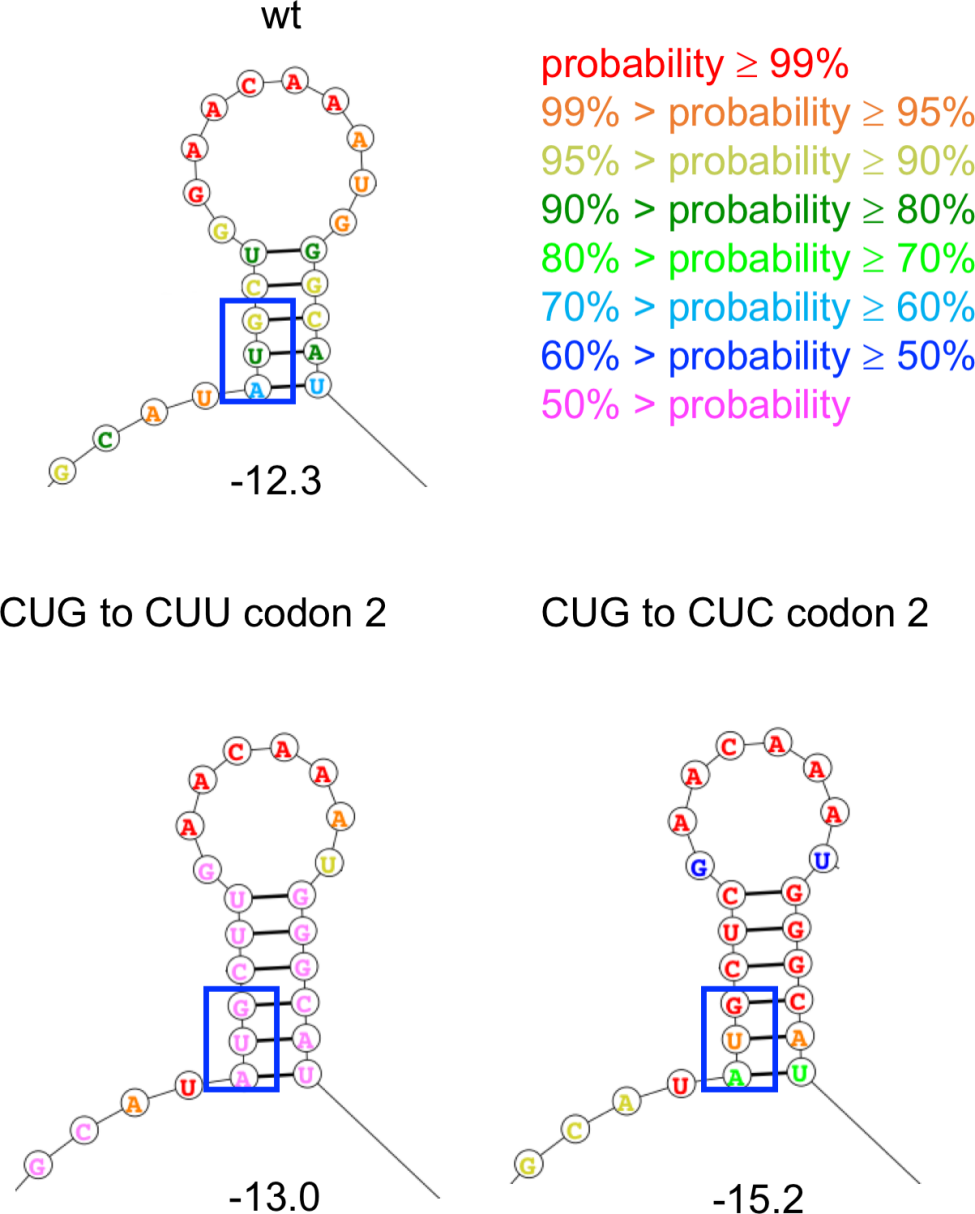
Predicted lowest-free energy structures of the region surrounding the AUG start codon (blue box) in wild-type and in mutant proA* transcripts in which synonymous mutations in codon 2 decrease growth rate. Numbers underneath the structures indicate the folding energy calculated by the Fold algorithm in kcal/mol.

**Fig 11:**
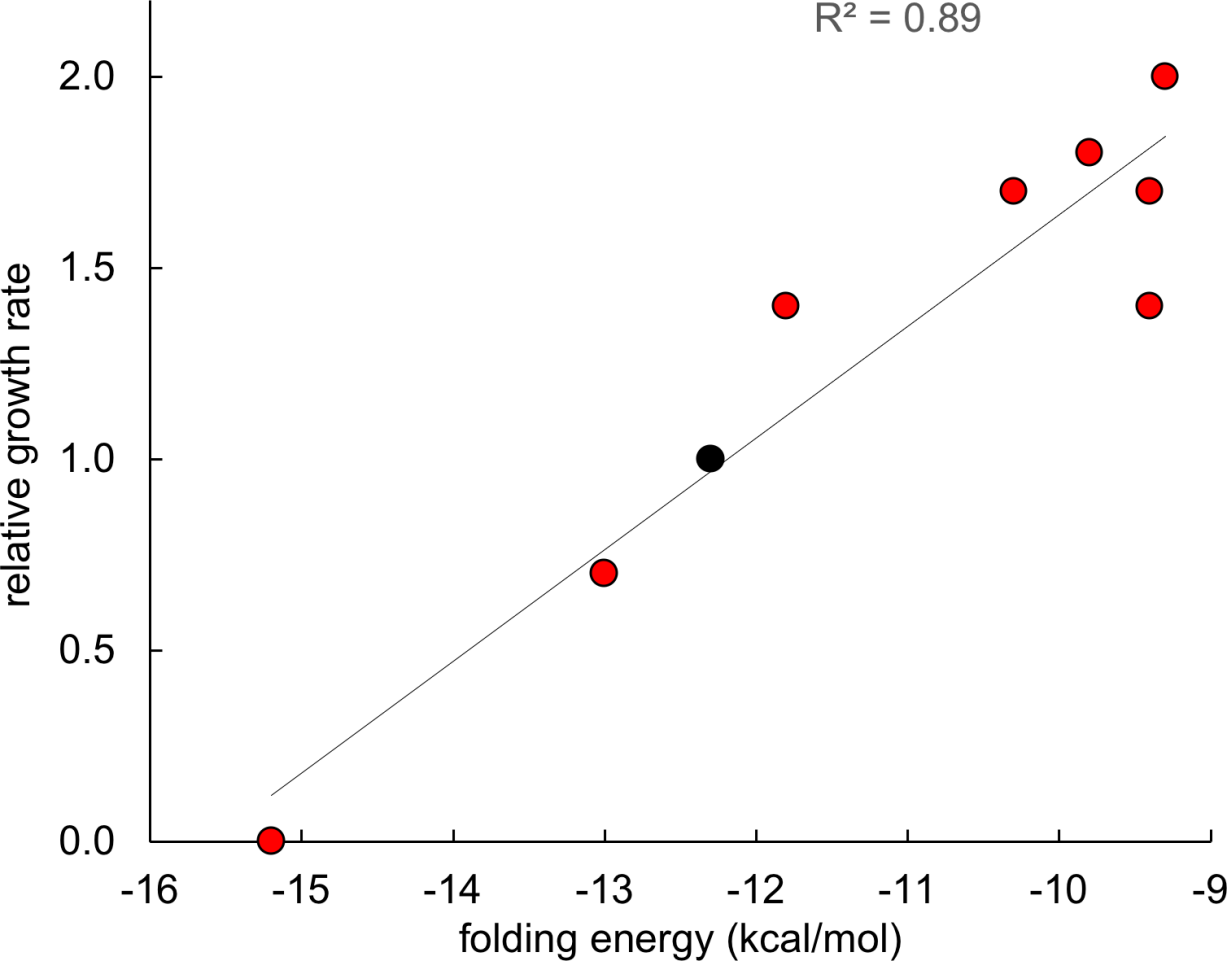
Growth rate is proportional to the stability of the 53-nt head region of the proA* region in the ΔargC proA* strain carrying the promoter mutation M1 for strains containing synonymous mutations in codons 2 and 6. Black dot indicates the strain that contains M1 only and has the wild-type sequence at the head of the proA* mRNA.

Fig 9 shows the effect of mutations M3, M4 and two of the synonymous mutations in codons 2 and 6 that increase growth rate on the lowest energy structure predicted by the Fold algorithm. M3 and M4 destabilize the structure by 0.5 and 2.0 kcal/mol, respectively, and decrease the probability that the nucleotides in the stem will be found in the depicted base-paired stem-loop structure. The effect of combining M3 and M4 is additive, suggesting that the effects of the two mutations are independent. A synonymous mutation in codon 2 that changes CUG to UUG weakens the stem by changing a GC base pair into a GU base pair. The most dramatic effects were seen for mutations in codon 6 that disrupt the middle base pair in the 5-base-pair stem. Changing GGC to GGG (Fig 9 and S3 Fig) or GGA (S3 Fig) is predicted to disrupt the stem-loop entirely, leading to a structure that contains a different stem-loop with only three base pairs. Equally dramatic effects are caused by synonymous mutations that increase the stability of the stem structure (Fig 10). A synonymous mutation that adds a GU base pair increases stability by 0.7 kcal/mol, and a mutation that adds a GC base pair increases stability by 2.9 kcal/mol. Notably, a strain carrying the latter mutation does not grow at all in M9/glucose. Fig 11 shows that growth rate is linearly related to the stability of the minimal free energy structure for this set of synonymous mutations in codons 2 and 6.

**Fig 12:**
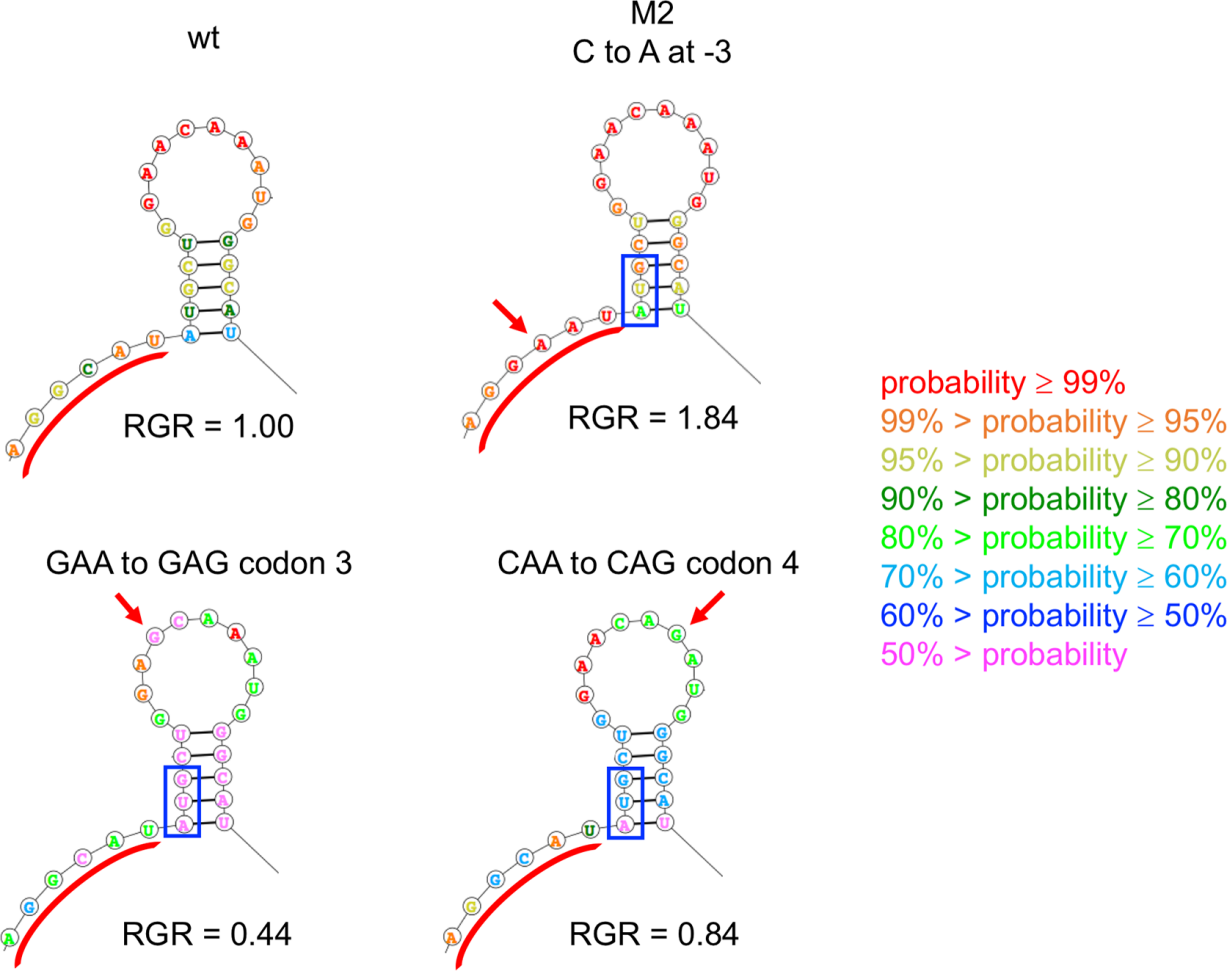
M2 and synonymous mutations in codons 3 and 4 affect the probability that the region preceding the start codon is single-stranded. The minimal folding energy of all four structures is -12.3 kcal/mol. RGR, relative growth rate.

In contrast to synonymous mutations in codons 2 and 6, the intergenic mutation M2 and synonymous mutations in codons 3 and 4 do not change the minimal folding energy (Fig 12). However, they have a striking effect on the probabilities that nucleotides preceding the start codon are single-stranded. The mutation at -3 (M2) increases the probability that the region preceding the start codon will be single-stranded and increases growth rate by 84%. In contrast, synonymous mutations in codons 3 and 4 decrease the probability that the region preceding the start codon will be single-stranded and decrease growth rate by 56 and 16%, respectively.

## Discussion

Deletion of *argC* requires *S*. *enterica* to recruit a promiscuous enzyme within its proteome to serve an essential function during growth on glucose. This system provides a good model for many situations in which a new enzyme is needed, such as the presence of a toxin or the availability of a new source of carbon, nitrogen or phosphorus. Recruitment of a mutant version of ProA solves the immediate problem of providing an enzyme that is newly required for growth. (Wild-type ProA cannot substitute for the missing ArgC.) This situation is particularly interesting because both the new and the original functions of the enzyme are required to support growth.

When growth of microbes is limited by the inefficiency of a weak-link bifunctional enzyme, selection favors emergence of mutants that have managed to increase the level of one or both growth-restricting activities. This is often accomplished, at least initially, by promoter mutations or gene amplification. Both mechanisms substantially improve growth rate in an ΔargC proA* strain of *E*. *coli* [9]. In the *Salmonella* ΔargC proA* strain, a point mutation in the -10 region of the promoter (TAAAAC to TAAAAT) increases its similarity to the sigma 70 consensus sequence (TATAAT) [23], increases expression of the proBA* operon by 11-fold, and increases growth rate by 9-fold. However, we did not observe gene amplification in either large colonies on plates or in four lineages that were adapted in glucose medium for 260 generations, a time by which massive amplification of the region surrounding the proBA* operon had occurred in *E*. *coli* [9]. The lack of amplification is surprising, as previous studies have shown that duplications (the precursors of amplifications) occur readily in many regions of the *S*. *enterica* genome [24]. Since gene duplication is the prerequisite for divergence of gene copies toward alleles encoding two specialist enzymes, the lack of duplication constrains the prospects for evolution of a new enzyme with specialized ArgC activity, as well as the potential for reversion of the proA* allele to restore the previous level of ProA activity. These results suggest that the abilities of even relatively closely related microbes to evolve a new enzyme in the face of an environmental challenge may be dramatically different.

The growth rate of the *Salmonella* ΔargC proA* strain is substantially lower than that of wild-type cells even after acquisition of a promoter mutation, so there is still selective pressure for mutations that improve the ability to make proline and arginine. We discovered only one beneficial non-synonymous mutation in proA* in the four adapted lineages. This mutation changes Glu34 to Gly. The corresponding residue in the crystal structure of *Thermatoga maritima* ProA (PDB 1O20) is over 30 Å away from the catalytic Cys residue (S4 Fig). Efforts to determine the effect of this mutation are in progress.

Striking improvements in fitness of the ΔargC proA* strain were also attained due to an intergenic mutation just upstream of the start codon (M2) and two synonymous mutations in the first six codons (M3 and M4). To explore the role of codon choice in this region of the proA* mRNA, we introduced every possible synonymous mutation into codons 2, 3, 4 and 6 in a background containing the M1 promoter mutation. These mutations had surprisingly large effects on fitness, ranging from a doubling of growth rate to complete inhibition of growth (Fig 7 and Table 3). Although six mutations in this region increased growth rate, we found only two in the adapted lineages. This discrepancy is likely due to the limited number of clones (seven derived from only four evolved lineages) whose genomes were sequenced. Further, three of the four mutations that were not found are transversions, which are 2-7-fold less common than transitions such as M3 and M4 [25], and the other is unlikely to occur over a short period of selection because it requires two point mutations.

Synonymous mutations might affect fitness via any of several mechanisms discussed in the introduction, including generation of a new promoter, alteration of mRNA stability and/or translation efficiency, binding of a small non-coding RNA to a mRNA, and alteration of protein folding due to changes in the tempo of translation. Since the synonymous mutations we found are within the body of the gene encoding the weak-link enzyme, they cannot have generated a new promoter for proA*. We can also dismiss effects on protein folding. The use of rare codons at specific points in a mRNA has been suggested to allow folding of translated polypeptide sequences in the absence of downstream sequences that might interfere with proper folding. We identified high-impact synonymous mutations within the first six codons. Any effects of translation rate on protein folding would be irrelevant in the initial six amino acids. There is insufficient space for folding in the ribosome exit tunnel until the chain has reached the last 20 Å of the exit tunnel, which requires translation of at least 20 amino acids [26, 27].

Several of the synonymous mutations and the mutation at -3 relative to the start codon (M2) had substantial effects on the level of proA* mRNA, and, not surprisingly, growth rate increased as a function of proA* mRNA level. The effects of these mutations on the levels of proA* mRNA might be due to effects on transcription, degradation, or both. The former is unlikely; mutations around the head of the proA* mRNA should not affect the rate at which the entire proBA* operon is transcribed because proA* is the second gene in the operon. Further, these mutations are too close to the start codon for proA* to generate a new promoter. Thus, the mutations most likely affect the rate of degradation of the proA* mRNA. These effects might be due to alterations in binding to a small regulatory RNA. However, of the 140 recognized small regulatory RNAs in *S*. *enterica* serovar Typhimurium, none is known to bind in the *proBA* operon [23]. A final possibility is that differences in degradation are due to the well-established link between mRNA degradation and translation efficiency. Ribosome occupancy protects mRNA from degradation by physically shielding the mRNA from endonucleases [28]. Ribosome binding also prevents premature transcription termination, which occurs when translation is not initiated promptly after the initial part of the transcript is produced by RNA polymerase [29].

Translation efficiency is determined by the efficiency of initiation [30], which depends on a host of factors, including the strength of the Shine-Dalgarno sequence, the strength of the start codon, the spacing between these elements, the nature of the codons in the head of the mRNA, and the accessibility of the region between the Shine-Dalgarno sequence and start codon. Biases in codon usage in the heads of mRNAs have been recognized since the 1980s [31, 32] and have been ascribed to either the importance of minimizing secondary structure in the region that must bind to the 30S ribosomal subunit to initiate ribosome assembly [31–33], or to the benefits of a slow ramp in translation speed due to rare codons at the beginning of transcripts (30–50 codons) that prevents ribosome traffic jams [34]. Both factors may be important, and cannot always be deconvoluted. In our case, every synonymous mutation except one–both beneficial and detrimental–changed a common codon into a rare codon (S4 Table), suggesting that the beneficial effects cannot be attributed to the latter mechanism.

Our modeling results suggest that the intergenic mutation at -3 and synonymous mutations in the first six codons alter the propensity for secondary structure around the Shine-Dalgarno sequence and start codon. This region must be single-stranded to bind to the 30S subunit of the ribosome prior to assembly of the full ribosome [35], as the 30S initiation complex does not contain a competent GTPase that can use energy to unwind secondary structures. (IF2, which is present in the initiation complex, is not activated to hydrolyze GTP until after the 50S subunit binds [36, 37].) Mutations that decrease the stability of the stem-loop that sequesters the start codon increase the level of proA* mRNA and growth rate by up to 2-fold. Other beneficial mutations do not affect the stability of the stem-loop structure, but increase the probability that the 10 nucleotides preceding the start codon will be single-stranded. In contrast, detrimental mutations either decrease the probability that the 10 nucleotides preceding the start codon will be single-stranded or increase the stability of the stem-loop structure that sequesters the start codon. Notably, a mutation that adds an extra GC base pair to the stem-loop structure is sufficient to prevent growth entirely.

**Fig 13:**
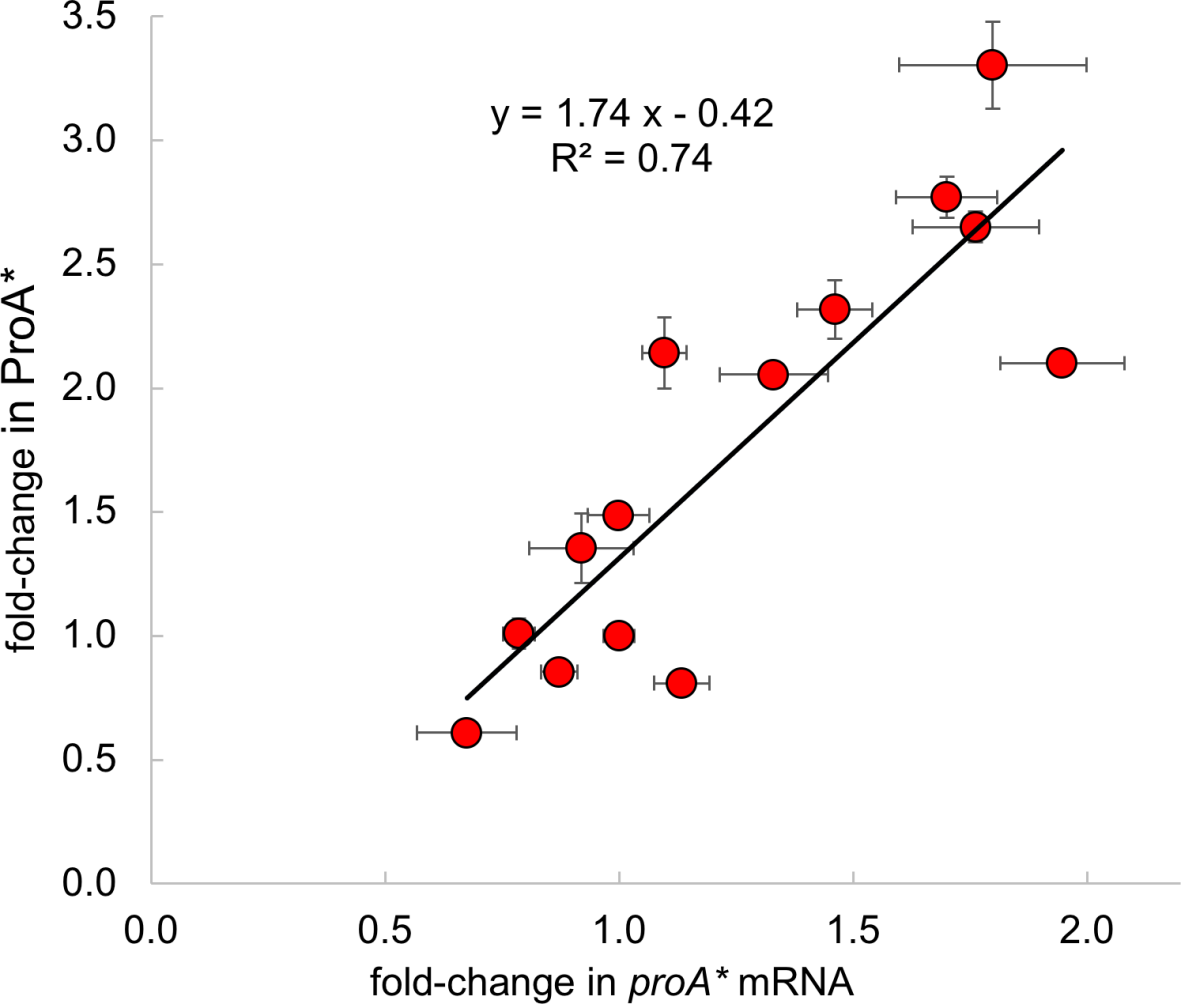
Fold-change in ProA* as a function of fold-change in the levels of proA* mRNA due to mutations in the head region of the proA* mRNA.

Our computational results support the hypothesis that synonymous mutations in the head region of the proA* mRNA affect translation efficiency. Experimental support for this notion is provided by the observation that the slope of a plot of fold-change in ProA* vs fold-change in proA* mRNA is 1.7 (Fig 13); if translation efficiency were unaffected by the mutations, the slope would be 1.0. This result indicates that changes in ProA* levels are due not only to changes in levels of proA* mRNA, but also to changes in the efficiency of translation.

Our results are consistent with recent computational studies showing that folding energies in 39-nucleotide windows in every mRNA encoded by 414 bacterial genomes are less negative around the start codon than in the rest of the transcripts; this decrease in stability is associated with the use of rare codons that tend to be AU-rich [38]. The importance of low secondary structure around the ribosome binding site was reinforced by a study of a library of recoded GFP variants in which synonymous mutations were introduced randomly throughout the gene (with an average of 114 differences between pairs of sequences). Expression was highest for sequences for which secondary structure was minimal around the ribosome binding site (30 nucleotides centered around the start codon) [39]. Finally, an analysis of >14,000 synthetic reporters in *E*. *coli* demonstrated that increased secondary structure in the first 120 nucleotides of a mRNA decreases translation efficiency [40].

Substantial increases in growth rate due to synonymous mutations in a weak-link enzyme have been reported in two other cases. Agashe et al. recoded the gene encoding formaldehyde activating enzyme (FAE), which is required for growth of *Methylobacterium extorquens* AM1 on methanol and methylamine, with 46–150 synonymous mutations. Every re-coded version caused a significant decrease in growth rate on methanol and in the level of FAE [41]. In a subsequent adaptive evolution experiment, growth of three strains carrying recoded versions of *fae* was substantially improved by synonymous mutations in codons 4, 9 and 13 [42]. Further analysis of a set of 37 mutants showed no correlation between growth rate and either the computed folding energies of a 100-nucleotide fragment surrounding the start codon or the affinity between the Shine-Dalgarno sequence and the anti-Shine-Dalgarno sequence on the 30S ribosomal subunit, suggesting that epistatic interactions within the widely different *fae* alleles influenced the effects of single synonymous mutations on fitness. In another interesting case, Bailey et al. discovered two synonymous mutations in codons 15 and 38 of a glucose permease in a *Pseudomonas fluorescens* population evolved in the presence of glucose for 1000 generations. The mechanistic basis for the beneficial effects of these synonymous mutations, which increased expression of the glucose permease and increased fitness by 7–9%, was not clear [16].

The results reported here indicate that synonymous mutations can have unexpectedly large effects on fitness when growth rate is limited by a weak-link enzyme. While it has been recognized for decades that codon choice can have substantial effects on the levels of the expressed proteins, most previous work has been directed at either understanding the mechanism of translation or at optimizing protein expression for biotechnological purposes. The magnitude of the fitness effects of synonymous mutations under strong selective pressures we have reported here suggests that synonymous mutations may have played a previously unappreciated role in adaptation of microbes to novel stresses, particularly when gene duplication/amplification is not advantageous. However, the mechanisms responsible for increasing fitness are clearly not the same in this study and those of Agashe et al. [42] and Bailey et al [16], suggesting that there is more to learn about the interplay between synonymous mutations and adaptive evolution, particularly under strong selection.

## Materials and methods

### Strains and culture conditions

*Salmonella enterica* subsp. *enterica serovar* Typhimurium *str*. SL1344 (hereafter *S*. *enterica*) was obtained from the Detweiler lab at the University of Colorado Boulder. Strains we derived by genome editing of this ancestral strain are listed in Table 2. *S*. *enterica* strains were grown in LB or M9 medium [43] containing 0.2% glucose supplemented with 100 μg/mL ampicillin (amp), 35 μg/mL chloramphenicol (chl), or 100 ng/mL anhydrotetracycline (atc) as required for selection of strains during genome editing. *S*. *enterica* is naturally resistant to streptomycin (strep), so streptomycin was added to media at a concentration of 50 μg/mL to minimize contamination with other bacteria. When proline and/or arginine were added to the medium, the final concentrations (0.4 mM proline and 5.4 mM arginine) correspond to those found in EZ-rich medium [44].

### Primers for PCR, qPCR, and RT-PCR

Primers (S2 Table) were ordered from IDT with standard desalting.

### Construction of the ΔargC proA* parental strain

A nonsense mutation in *hisG* in the ancestral strain of *S*. *enterica* was reversed using a genome editing method developed in our laboratory [45]. The same method was used to delete *argC* and change the GAA codon specifying Glu382 to GCA specifying Ala to generate the parental strain for this work. We performed these editing steps in two biological replicates. Whole genome sequencing indicated that the replicated were identical, and therefore both are designated JK328.

### Screening for improved growth of the ΔargC proA* strain on plates containing M9/glucose

Fifty-μL aliquots of freezer stocks of JK328 were streaked onto 15 plates containing LB/strep and the plates were incubated at 37°C overnight. A single colony from each plate was washed twice with 100 μL sterile PBS, suspended in 100 μL sterile PBS, and diluted 1:10,000. Fifty-μL aliquots of each suspension were spread onto 15 plates containing M9/glucose/strep and the plates were incubated at 37°C for 5–6 days. One hundred colonies that were larger than average were suspended in 20 μL sterile H_2_O. Each suspension was streaked onto plates containing M9/glucose/strep and the plates were incubated for two days at 37°C. Genomic DNA was prepared from a single colony from each of the 100 colonies that had been streaked onto the isolation plates. The copy number of the proBA* operon was determined by qPCR for all 100 colonies as described below; the entire proBA* operon was sequenced in 50 colonies using Sanger sequencing (Macrogen).

### Evolution of the ΔargC proA* strain in M9/glucose liquid medium

Four starter cultures of 2 mL LB/strep were inoculated with individual colonies of the parental strain JK328 and the cultures were incubated at 37°C with shaking. Cultures were harvested at mid-log phase by centrifugation at 10,000 x g for 1.5 min at room temperature. The pellets were washed twice with 1 mL ice-cold sterile PBS and then suspended in 1 mL ice-cold sterile PBS. Aliquots of each suspension were inoculated into 5 mL M9/glucose/strep to give an initial OD_600_ of 0.001 and the four parallel cultures were incubated at 37°C with shaking. The change in OD_600_ by the next day was used to calculate the number of generations that had occurred during growth of the culture. At approximately mid-log phase, aliquots of each culture were transferred to fresh medium to give an initial OD_600_ = 0.001. (On two occasions after growth rate improved, the initial OD was adjusted to 0.0001 or 0.00001 to ensure that the cultures would not reach stationary phase before the next day. After that point, dilutions were carried out to an initial OD_600_ of 0.001 twice a day.) At each transfer, one mL of each culture was used to make a freezer stock by adding DMSO to 10% (v/v)) and one mL was harvested via centrifugation at 10,000 x g for 1.5 min at room temperature. The cell pellets were stored at -20°C for later preparation of genomic DNA for Sanger sequencing and/or qPCR. The evolution experiment was carried out for one month (approximately 260 generations).

### Construction of mutant strains

Construction of *S*. *enterica* strains containing mutations in the proAB* operon using the methods described by Kim et al. [45] is described in the Supporting Material.

### Measurement of growth rates

Aliquots of freezer stocks were streaked onto plates containing LB/strep and grown overnight at 37°C. Single colonies were used to inoculate 2 mL LB and grown to early log-phase at 37°C. One mL aliquots were harvested via centrifugation at 4,500 x g for 8 min at room temperature. Pellets were washed four times with 1.0 mL sterile PBS and re-suspended in 500 μL M9/glucose. The cells were diluted to an OD_600_ of 0.01 in M9/glucose and 10 μl aliquots were used to inoculate wells in a 96-well plate containing 90 μL M9/glucose to give an initial OD_600_ of 0.001. The plates were incubated in a Varioskan (Thermo) plate reader at 37°C with shaking every 5 minutes. The absorbance at 600 nm was measured every 20 minutes for up to 500 hours. The baseline absorbance for each well (the average over several smoothed data points before growth) was subtracted from each point of the smoothed growth curve. Growth parameters (maximum specific growth, μ_max_; lag time, λ; maximum growth, A_max_) were estimated by non-linear regression using the modified Gompertz equation [46]. Non-linear least-squares regression was performed in Excel using the Solver feature.

### Whole-genome sequencing

We sequenced the whole genomes of adapted clones and reconstructed strains to identify mutations in the proBA* operon as well as elsewhere in the genome that might contribute to fitness. Genomic DNA was extracted from overnight cultures using the Invitrogen PureLink Genomic DNA mini kit. Libraries were prepared using a modified Illumina Nextera protocol and multiplexed onto a single run on an Illumina NextSeq500 to produce 151-bp paired-end reads [47]. These resulted in coverage of the ancestral *S*. *enterica str*. SL1344 genome ranging from 50 to 200. Reads were trimmed using BBtools v35.82 (DOE Joint Genome Institute) and mapped using breseq v0.28.1 [48].

### Determination of proA* copy number

Procedures for determination of proA* copy number by qPCR are described in the Supporting Material.

### Analysis of transcript levels by RT-qPCR

Procedures for analysis of transcript levels by RT-qPCR are described in the Supporting Material.

### Analysis of ProA* and ProB levels by label-free proteomics

Procedures for analysis of ProA* and ProB levels by label-free proteomics are described in the Supporting Material.

## Supporting information

S1 Text Supporting methods. (DOCX)

S1 Fig Large colonies observed against a background of small colonies when the ΔargC proA* strain is spread on plates containing M9/glucose. (PDF)

S2 Fig The synonymous mutations M3 and M4 have negative epistatic interactions with the promoter mutation M1. The predicted effects for the case of no epistasis were obtained by multiplying the fold increases due to each mutation alone. The combination of M1 and M4 would be expected to produce a strain that is more fit than the wild-type, so the predicted growth rate μ should in reality be equivalent to that of the wild-type strain. Strain designations: WT, JK411; parental strain, JK328; M1, JC559; M3, WK014; M1 + M3, JC596; M4, WK012; M1 + M4, JC592 (see Table 2 in main text). WK012 and WK014 were constructed from JC559 by reversion of the promoter mutation M1 and introduction of either M3 or M4. WK014 acquired a point mutation that changes Gly95 to Ser in a putative bacteriophage protein (SL1344_2574) during strain construction that should not affect fitness.
(PDF)

S3 Fig Lowest-free energy structures predicted by the Fold algorithm for 53-nt fragments of the head region of proA* transcripts with mutations in codon 6. Only the region surrounding the AUG start codon is shown. Numbers underneath the structures indicate the folding energy calculated by the Fold algorithm in kcal/mol.
(PDF)

S4 Fig The position of the lysine residue (blue) in *Thermatoga maritima* ProA corresponding to Glu34 in *S. enterica* ProA, which is changed to Gly by M5. The active site cysteine is shown in yellow to mark the active site. This figure was made using the UCSF Chimera package [49]. Chimera is developed by the Resource for Biocomputing, Visualization, and Informatics at the UCSF Chimera package. Chimera is developed by the Resource for Biocomputing, Visualization, and Informatics at the University of California, San Francisco (supported by NIGMS P41-GM103311).
(PDF)

S1 Table Peptide counts for ProB and ProA* in all strains. (XLSX)

S2 Table Primers used for PCR, qPCR and RT-PCR. (DOCX)

S3 Table Efficiencies of primer sets for qPCR and RT-qPCR. (DOCX)

S4 Table Codons and corresponding codon frequencies for synonymous mutations in codons 2–6 of proA*. (DOCX)
