# Supplementary material for: Synonymous mutations make dramatic contributions to fitness when growth is limited by a weak-link enzyme
Source: PLoS Genet. 2018 Aug 27;14(8):e1007615. doi: 10.1371/journal.pgen.1007615 (PMC6128649; doi:10.1371/journal.pgen.1007615)
Supplement: S1 Text — (DOCX) [file pgen.1007615.s001.docx]

**Supporting methods**

Genome editing procedures

Mutations to correct the histidine auxotrophy of the *Salmonella enterica* subsp. *enterica serovar* Typhimurium *str.* SL1344 and to introduce various mutations into the genome of the parental strain that lacks *argC*  and carries the *proA** allele were carried out using the genome editing procedure of Kim et al. (1).

Mutation cassettes were designed to include two mutation fragments flanking the *cat* gene conferring chloramphenicol resistance. The *cat* gene was preceded by a double terminator and an I-SceI cleavage site. Homology regions HR1 and HR2 (~100 bp each) upstream and downstream, respectively, of the *cat* gene were used to target the mutation cassette to a specific site in the genome. The 3’-end of HR1 and the 5’-end of HR2 contained an ~30 bp overlapping region (HR3) that included the desired mutation. The mutation fragments (~250 bp) were either amplified by PCR from genomic DNA isolated from mutants collected during adaptation experiments or ordered as gBlocks from IDT. Primers with ~20 bp overhangs were used to amplify the mutation fragments and the *cat* selection marker for subsequent assembly into a pUC19 vector backbone containing a gene conferring ampicillin resistance. The linearized pUC19 vector backbone, mutation fragments, and *cat* selection marker were gel-purified using a Thermoscientific gel extraction kit and quantified by Qubit HS dsDNA kit (Invitrogen) prior to assembly. Gibson Assembly (NEB) (2) was done according to the manufacturer’s protocol using the linearized pUC19 backbone, 5’-mutation fragment, 3’-mutation fragment, and *cat* selection cassette at molar ratios of 1:5:5:3. The resulting mutation-cassette template plasmids could then be used for subsequent preparation of mutation cassettes for multiple experiments.

Mutation cassettes were amplified from mutation-cassette template plasmids by PCR using primers that bind within the 5’- and 3’-mutation fragments and then gel-purified using a Thermoscientific gel-extraction kit (Thermoscientific). The amplified mutation cassette fragments were treated with Dpn-1 (NEB) to remove residual plasmid template, gel-purified, and then quantified using Qubit HS dsDNA kit (Invitrogen).

Insertion of the mutation cassette in the targeted genomic site was accomplished using the helper plasmid pSLTS, which carries the genes encoding I-SceI under control of the tetracycline promoter, the lambda-Red recombination genes under control of the P_BAD_ arabinose promoter, and an *amp* gene conferring ampicillin resistance. After transformation with pSLTS and the mutation cassette by electroporation, cells were spread onto plates containing LB/amp/chl and incubated overnight at 30 ˚C to select for colonies that had acquired pSLTS and had also incorporated the *cat* selection marker into the genome. Colonies were then spread onto plates containing LB/amp/atc and incubated overnight at 30 ˚C. Atc induces expression of I-Sce1 endonuclease, which cleaves at a site within the mutation cassette. Cells that repair the break via recombination of the overlapping HR3 segments flanking the *cat* gene survive, with concomitant loss of the *cat* gene itself. Colonies from plates containing LB/amp/atc were patched onto plates containing LB/amp/chl and LB/amp to identify those that had lost the *cat* selection marker. Correct introduction of the intended genetic change was confirmed by Sanger sequencing. The pSLTS plasmid (which contains a temperature-sensitive origin of replication) was then removed by incubation at 37 ˚C overnight.

Analysis of Copy Number by qPCR

Primer3Plus (https://primer3plus.com/) was used to design three primer sets for *proA* and the reference genes *icdA* and *gyrB*, which we assumed to be present in a single copy. The following settings were used: Na^+^, 50 mM; Mg^++^, 1.5 mM; dNTPs, 0.6 mM; target type, DNA; and oligo concentration, 0.25 μM. mFold (3) was used to estimate the ΔG of secondary structures in the amplified product that could interfere with primer binding. Settings for mFold were the same as those for primer design with Primer3Plus. IDT Oligo-analyzer (https://www.idtdna.com/calc/analyzer) was used to estimate the ΔG of self-dimerization and hetero-dimerization to ensure that the primers would not anneal to each other and produce faulty primer efficiencies.

The efficiency of each primer set was evaluated by generating a standard curve using 1:10 serial dilutions of genomic DNA from the parental strain JK328. Two µL of each dilution were used in triplicate with 0.4 µL each of the 10 µM forward and reverse primers, 7.2 µL sterile H_2_O, and 10 µL 2X SYBR Green master mix (Applied Biosystems). Reaction mixtures without template for each primer set were included during each run to check for contamination. Cycling conditions for a standard run with Power SYBR Green in a 7500 Fast Real-Time PCR system (Applied Biosystems) were as follows: 95 °C for 10 min; 40 cycles of 95 °C for 15 s, 60 °C for 60 s. A melt curve was performed immediately following qPCR in order to test for multiple products or sample contamination. Baseline adjustment and thresholds were manually set in the 7500 StepOne v2.3 software. Primer sets with the highest efficiencies (Supplementary Table 2) were selected for identification of *proA** copy number in adapted clones.

qPCR to assess the copy number of the *proBA** operon was carried out using genomic DNA under the conditions described above and using the parental strain (JK328) as an inter-run calibrator and *icdA* and *gyrB* as internal reference genes. No-template controls were included to check for contamination. Primer efficiencies and Cq values for each reaction were exported into Excel for analysis with qBase in order to generate the normalized relative quantity (NRQ) of *proA** for each mutant (4).

Analysis of Transcript Levels by RT-qPCR

Growth of starter cultures from freezer stocks was carried out as described in the text. Culture tubes containing 4.9 mL M9/glucose were inoculated to give an initial OD_600_ of 0.001. Cultures were grown in triplicate at 37 °C with shaking at 220 revolutions per minute to mid-log phase (OD_600_ 0.2-0.4, 1-5 days). One-mL aliquots of each culture were harvested by centrifugation at 16,000 xg and 4 °C for 1 min and treated with RNAprotect (Qiagen) before storage at -68 °C. Frozen pellets were thawed and the RNA purified using RNeasy spin columns (Qiagen) according to the manufacturer’s protocol. An additional DNase treatment was performed using Turbo DNase (Life Technologies) according to the manufacturer’s protocol. RNA concentration was determined using the Qubit RNA HS assay kit with a Qubit 3.0 fluorometer (Life Technologies). Reverse transcription was performed with 8-100 ng RNA using Superscript VILO Mastermix (Invitrogen) according to the manufacturer’s protocol except that the duration of the 42° C incubation step was increased to 2 hours. Aliquots of the cDNA obtained from these reactions were stored at -20 °C.

The primer sets used for *proA**, *icdA*, and *gyrB* in the qPCR analyses described above were also used for RT-qPCR. The same method used to design these primer sets was used to design a primer set for *proB*. *gyrB*, which encodes a subunit of DNA gyrase, has been determined to be one of the most invariably expressed genes in a recent review of bacterial reference genes (5).  *icdA*, which encodes isocitrate dehydrogenase, has been used previously to normalize for gene expression in the marine bacterium *Zobellia galactanivorans* (6). The constancy of *gyrB* and *icdA* expression levels was verified across all samples using geNorm (4). Stability M-value and coefficient of variation were determined to be 0.22 and 23.8, respectively. Cycling conditions for RT-qPCR were as follows: 95 °C for 10 min; 40 cycles of 95 °C for 15 s, 60 °C for 60 s. A melt curve was performed immediately following RT-qPCR to test for multiple products or contamination of the samples.

Analysis of ProA* and ProB Levels by Label-Free Mass Spectrometry

Samples were prepared for mass spectrometry analyses using the filter-aided sample preparation (FASP) method (7). Briefly, cell pellets were solubilized in 4% (w/v) SDS, 10 mM tris(2-carboxyethyl)phosphine (TCEP) and 40 mM 2-chloroacetamide in 0.1 M Tris-HCl, pH 8.5, boiled for 10 minutes, and then processed in a Bioruptor (Diagenode) for 10 cycles of 30 seconds on and 30 seconds off at 20 ⁰C. The samples were then diluted 10-fold with 8 M urea in 0.1 M Tris-HCl, pH 8.5, and loaded into an Amicon Ultra ultrafiltration device (0.5 mL, 30 kD NMWL cutoff, Millipore). The samples were washed three times in the filters with 8 M urea in 0.1 M Tris-HCl, pH 8.5, and then three times with 2 M urea in 0.1 M Tris-HCl, pH 8.5. Endoproteinase Lys-C (Wako) was added at a ratio of 1:100 (by weight) and the sample was incubated for 2 hours with rocking at room temperature. At that point, trypsin (Pierce), also at a 1:100 ratio (by weight), was added and incubation was continued overnight with rocking at room temperature. Peptides were collected via centrifugation through the filter and desalted using an Oasis HLB cartridge (Waters) according to the manufacturer’s protocol. The solvent was removed by evaporative centrifugation using a SpeedVac (Thermo).

The resulting peptide samples were suspended in 3% (v/v) acetonitrile/0.1% (v/v) trifluoroacetic acid to a concentration of approximately 500 ng/mL and 1 µL was injected directly onto an M-class C18 column (1.7 µm, 130 Å, 75 µm X 250 mm, Waters) on a Waters M-class UPLC. Peptides were eluted into an Orbitrap Fusion mass spectrometer (Thermo Scientific) at 300 nL/minute using a gradient from 3% acetonitrile to 20% acetonitrile over a period of 100 minutes. Precursor mass spectra (MS1) were acquired with a resolution setting of 120,000 from m/z = 380 to m/z = 1500 with an automated gain control (AGC) target of 2.0 x 10^5^ and a maximum injection time of 50 ms. Dynamic exclusion time was set to 20 seconds with a mass tolerance of +/- 10 ppm. Peptides with m/z within +/- 0.8 Da of the parent ion were accumulated in the quadrupole prior to sequencing with a 3-second cycle time. All MS2 sequencing was performed by fragmenting peptides using higher-energy collision dissociation at 35% collision energy; fragment ions were detected in the linear ion trap. An AGC target of 1.0 x 10^4^ and a 35-second maximum injection time were used. Raw files were searched against the Uniprot Salmonella database SL1344 (downloaded December 18, 2017) using MaxQuant version 1.6.1.0 (8) with cysteine carbamidomethylation as a fixed modification. Methionine oxidation and protein N-terminal acetylation were searched as variable modifications. Relative protein abundance estimates were extracted as label-free quantitative (LFQ) values from the raw files using MaxQuant.

1. Kim J, Webb AM, Kershner JP, Blaskowski S, Copley SD. A versatile and highly efficient method for scarless genome editing in *Escherichia coli* and *Salmonella enterica*. BMC Biotechnol. 2014;14:84.

2. Gibson DG, Young L, Chuang RY, Venter JC, Hutchison CA, 3rd, Smith HO. Enzymatic assembly of DNA molecules up to several hundred kilobases. Nat Methods. 2009;6(5):343-5.

3. Zuker M. Mfold web server for nucleic acid folding and hybridization prediction. Nucleic Acids Res. 2003;31(13):3406-15.

4. Hellemans J, Mortier G, De Paepe A, Speleman F, Vandesompele J. qBase relative quantification framework and software for management and automated analysis of real-time quantitative PCR data. Genome Biol. 2007;8(2):R19.

5. Rocha DJ, Santos CS, Pacheco LG. Bacterial reference genes for gene expression studies by RT-qPCR: survey and analysis. Antonie Van Leeuwenhoek. 2015;108(3):685-93.

6. Thomas F, Barbeyron T, Michel G. Evaluation of reference genes for real-time quantitative PCR in the marine flavobacterium *Zobellia galactanivorans*. J Microbiol Methods. 2011;84(1):61-6.

7. Wisniewski JR. Quantitative evaluation of filter aided sample preparation (FASP) and multienzyme digestion FASP Protocols. Anal Chem. 2016;88(10):5438-43.

8. Tyanova S, Temu T, Cox J. The MaxQuant computational platform for mass spectrometry-based shotgun proteomics. Nat Protoc. 2016;11(12):2301-19.

9. Pettersen EF, Goddard TD, Huang CC, Couch GS, Greenblatt DM, Meng EC, et al. UCSF Chimera--a visualization system for exploratory research and analysis. J Comput Chem. 2004;25(13):1605-12.
