# Supplementary figures and images for: Synonymous mutations make dramatic contributions to fitness when growth is limited by a weak-link enzyme
Source: PLoS Genet. 2018 Aug 27;14(8):e1007615. doi: 10.1371/journal.pgen.1007615 (PMC6128649; doi:10.1371/journal.pgen.1007615)

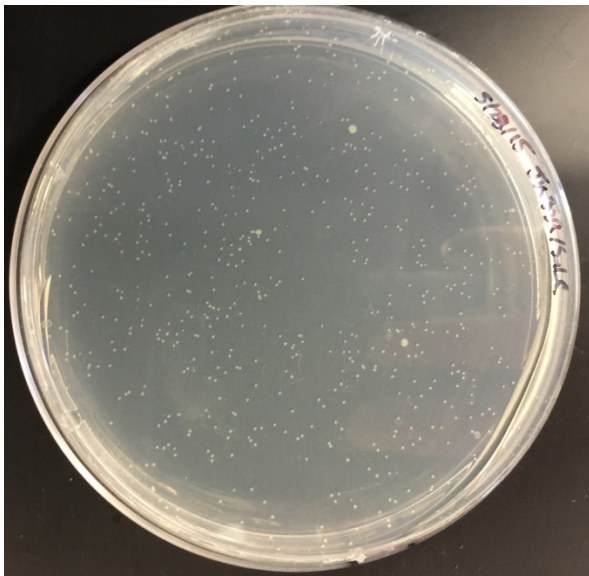

Supplement: S1 Fig — (PDF) [file pgen.1007615.s002.pdf]

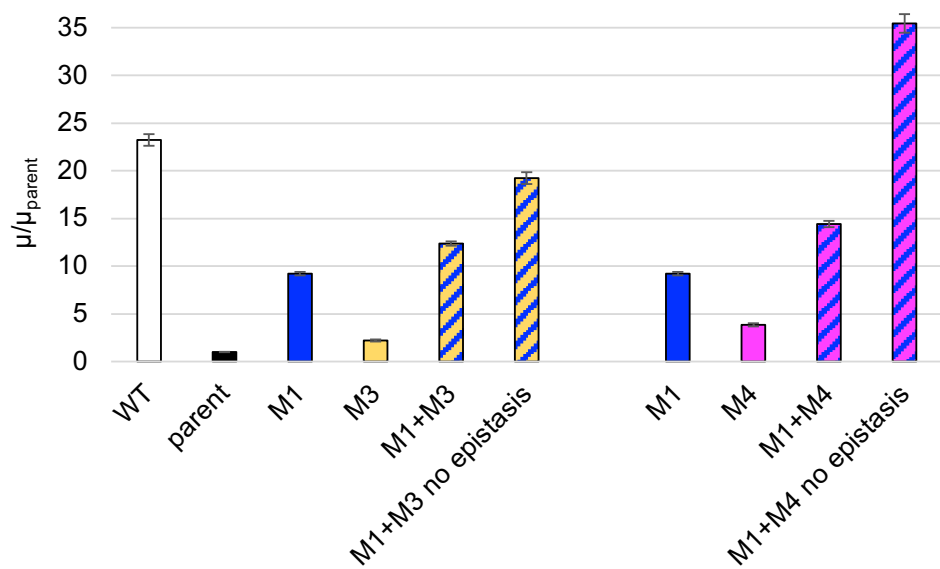

Supplement: S2 Fig — The predicted effects for the case of no epistasis were obtained by multiplying the fold increases due to each mutation alone. The combination of M1 and M4 would be expected to produce a strain that is more fit than the wild-type, so the predicted growth rate μ should in reality be equivalent to that of the wild-type strain. Strain designations: WT, JK411; parental strain, JK328; M1, JC559; M3, WK014; M1 + M3, JC596; M4, WK012; M1 + M4, JC592 (see Table 2 in main text). WK012 and WK014 were constructed from JC559 by reversion of the promoter mutation M1 and introduction of either M3 or M4. WK014 acquired a point mutation that changes Gly95 to Ser in a putative bacteriophage protein (SL1344_2574) during strain construction that should not affect fitness. (PDF) [file pgen.1007615.s003.pdf]

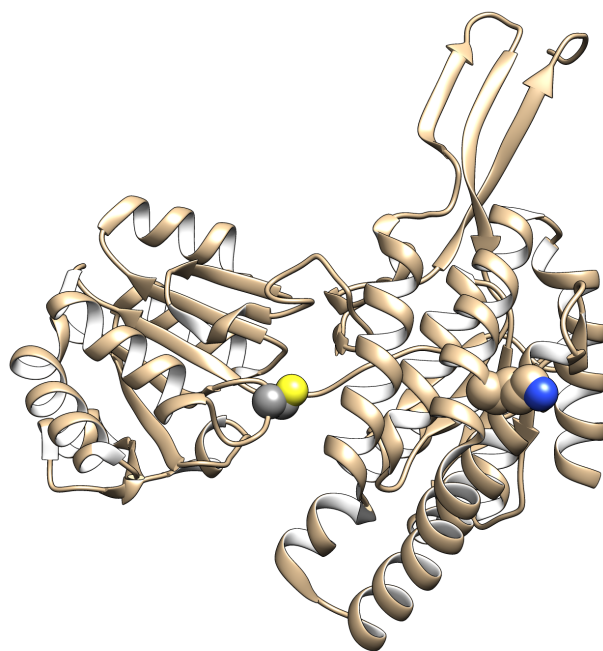

Supplement: S4 Fig — The active site cysteine is shown in yellow to mark the active site. This figure was made using the UCSF Chimera package [49]. Chimera is developed by the Resource for Biocomputing, Visualization, and Informatics at the UCSF Chimera package. Chimera is developed by the Resource for Biocomputing, Visualization, and Informatics at the University of California, San Francisco (supported by NIGMS P41-GM103311). (PDF) [file pgen.1007615.s005.pdf]
