# Supplementary material for: Synonymous mutations make dramatic contributions to fitness when growth is limited by a weak-link enzyme
Source: PLoS Genet. 2018 Aug 27;14(8):e1007615. doi: 10.1371/journal.pgen.1007615 (PMC6128649; doi:10.1371/journal.pgen.1007615)
Supplement: S3 Fig — Only the region surrounding the AUG start codon is shown. Numbers underneath the structures indicate the folding energy calculated by the Fold algorithm in kcal/mol. (PDF) [file pgen.1007615.s004.pdf]

codon 6 – GGC to GGG

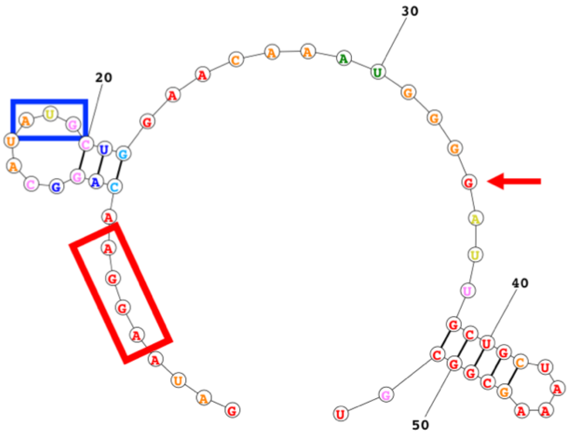

energy = -9.4

codon 6 – GGC to GGA

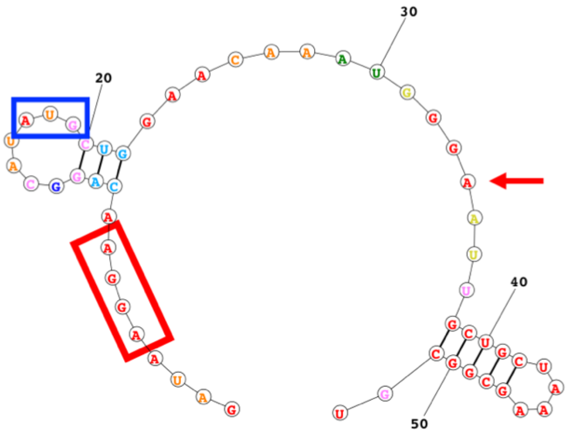

energy = -9.4

probability ≥ 99%
99% > probability ≥ 95%
95% > probability ≥ 90%
90% > probability ≥ 80%
80% > probability ≥ 70%
70% > probability ≥ 60%
60% > probability ≥ 50%
50% > probability
