# Supplementary material for: Synonymous mutations make dramatic contributions to fitness when growth is limited by a weak-link enzyme
Source: PLoS Genet. 2018 Aug 27;14(8):e1007615. doi: 10.1371/journal.pgen.1007615 (PMC6128649; doi:10.1371/journal.pgen.1007615)
Supplement: S2 Table — (DOCX) [file pgen.1007615.s007.docx]

**S2 Table**. Primers used for PCR, qPCR and RT-PCR.

| **primer name** | **direction** | **sequence (5'->3')** | **used for** | **amplicon location** |
| --- | --- | --- | --- | --- |
| JC-P11 | forward | GCGTCCGAATGTTACCGTTG | *proA*; qPCR and RT-qPCR | 367270-367344 |
| JC-P12 | reverse | TTCCCACCGCGTAGAATCAC | *proA*; qPCR and RT-qPCR | 367270-367344 |
| JC-P5 | forward | CAGGCGGAGCTTATCAAAGA | *proB*; RT-qPCR | 366343-366450 |
| JC-P6 | reverse | CCTGAAGCTTAGTGCTCATACC | *proB*; RT-qPCR | 366343-366450 |
| JC-P17 | forward | CGCCTGTATGAACCTGAACG | *icdA*; qPCR and RT-qPCR | 1282134-1282205 |
| JC-P18 | reverse | GCAATACCGATACCGCCAAC | *icdA*; qPCR and RT-qPCR | 1282134-1282205 |
| JC-P19 | forward | TGCCCGTGAAGGTCTGATTG | *gyrB*; qPCR and RT-qPCR | 4061524-4061642 |
| JC-P20 | reverse | TCATCTGCTGTTCTACCGCC | *gyrB*; qPCR and RT-qPCR | 4061524-4061642 |
