# Supplementary material for: Synonymous mutations make dramatic contributions to fitness when growth is limited by a weak-link enzyme
Source: PLoS Genet. 2018 Aug 27;14(8):e1007615. doi: 10.1371/journal.pgen.1007615 (PMC6128649; doi:10.1371/journal.pgen.1007615)
Supplement: S3 Table — (DOCX) [file pgen.1007615.s008.docx]

**S3 Table.** Efficiencies of primer sets for qPCR and RT-qPCR.

| gene | primers | efficiency for qPCR | efficiency for RT-qPCR |
| --- | --- | --- | --- |
| *proA* | JC-P11 and JC-P12 | 1.99 | 2.00 |
| *proB* | JC-P5 and JC-P6 |  | 2.00 |
| *icdA* | JC-P17 and JC-P18 | 1.97 | 2.00 |
| *gyrB* | JC-P19 and JC-P20 | 1.99 | 2.00 |
