# Supplementary material for: Synonymous mutations make dramatic contributions to fitness when growth is limited by a weak-link enzyme
Source: PLoS Genet. 2018 Aug 27;14(8):e1007615. doi: 10.1371/journal.pgen.1007615 (PMC6128649; doi:10.1371/journal.pgen.1007615)
Supplement: S4 Table — (DOCX) [file pgen.1007615.s009.docx]

**S4 Table.** Codons and corresponding codon frequencies for synonymous mutations in codons 2-6 of *proA**.^a^

| codon | amino acid | wild-type  codon (% usage) | mutant  codon (% usage) |
| --- | --- | --- | --- |
| beneficial mutations | | | |
| 2 | Leu | CUG (46) | UUG (12)  UUA (13)  CUA (5) |
| 4 | Gly | GGC (41) | GGU (28) |
| 6 | Gly | GGC (41) | GGU (28)  GGG (16)  GGA (15) |
| detrimental mutations | | | |
| 2 | Leu | CUG (46) | CUU (13)  CUC (11) |
| 3 | Glu | GAA (63) | GAG (37) |
| 4 | Gln | CAA (29) | CAG (71) |

**^a^** Codon frequencies from http://www.kazusa.or.jp/codon/cgi-bin/showcodon.cgi?species=602.
